# Genomic insights into multidrug resistance in clinical *Escherichia albertii*: plasmid coexistence, *intI1* prevalence, and interspecies dissemination risk

**DOI:** 10.3389/fmicb.2026.1778797

**Published:** 2026-02-24

**Authors:** Peihua Zhang, Xi Yang, Qian Liu, Xinxia Sui, Wang Zhang, Yanwen Xiong

**Affiliations:** 1 National Institute for Communicable Disease Control and Prevention, Chinese Center for Disease Control and Prevention, Beijing, China; 2 Department of Clinical Laboratory, Beijing Anzhen Hospital, Capital Medical University, Beijing, China; 3 The Third Affiliated Hospital of Zhengzhou University, Zhengzhou, Henan, China; 4 Hebei Key Laboratory of Intractable Pathogens, Shijiazhuang Center for Disease Control and Prevention, Shijiazhuang, Hebei, China

**Keywords:** antimicrobial resistance, class I integron, conjugative plasmids, *Escherichia albertii*, plasmid evolution

## Abstract

**Background:**

*Escherichia albertii* (*E. albertii*) is an emerging foodborne pathogen of growing clinical significance and increasing multidrug resistance (MDR). This study characterized the multidrug-resistant clinical strain *E. albertii* ESA311 to uncover the genetic basis of its resistance and the transmission potential of its mobile genetic elements.

**Methods:**

We performed whole-genome sequencing on strain ESA311 to identify plasmids, resistance genes, and virulence factors. Conjugation experiments were conducted to evaluate plasmid transferability. Phylogenetic analysis of the MDR plasmids elucidated their evolutionary history and geographical distribution. The prevalence of *intI1* and its correlation with MDR were analyzed across 160 clinical *E. albertii* isolates.

**Results:**

Whole-genome sequencing identified five plasmids coexisting in ESA311, with pESA311_1 and pESA311_2 harboring diverse antimicrobial resistance genes (ARGs) conferring resistance to seven antibiotic classes, facilitated by mobile genetic elements including insertion sequences (ISs) and a class 1 integron (*intI1*). Conjugation assays revealed a stable co-transfer consortium of pESA311_1, pESA311_2, and pESA311_5, driving concurrent dissemination of multidrug resistance and virulence (sporadically co-mobilize of pESA311_4) and posing a co-selection risk. Further phylogenetic analysis identified homologous plasmids in other species, such as *Salmonella enterica* and *Escherichia coli*. Whereas the pESA311_1 lineage is largely restricted to China, pESA311_2 homologs have dispersed more broadly across different regions. In a broader surveillance of 160 clinical *E. albertii* isolates, *intI1* prevalence was 19.8% and strongly correlated with MDR.

**Conclusion:**

Our findings establish plasmids and *intI1* as pivotal drivers of MDR in *E. albertii*, and highlight the associated risks of resistance-virulence co-selection and interspecies plasmid dissemination.

## 1 Introduction

*Escherichia albertii* (*E. albertii*), an emerging member of the *Enterobacteriaceae* family, has increasingly been recognized as a significant foodborne pathogen in recent years (Gomes et al., 2020). Consistent with its key virulence factors—including intimin, cytolethal distending toxin, and Shiga toxin—*E. albertii* predominantly causes gastrointestinal disorders in humans (Muchaamba et al., 2022; Liu et al., 2023b). Since its definition by Huys et al. (2003), the spectrum of its clinical presentations has broadened from common gastroenteritis (Ooka et al., 2013; Masuda et al., 2020; Bengtsson et al., 2023) to less frequent but more severe extra-intestinal infections such as urinary tract infections and bacteremia (Afshin et al., 2020; Liu et al., 2023a), reflecting a steady ascent in its hazard level. In addition to sporadic infectious cases reported across various geographical regions (Muchaamba et al., 2022), *E. albertii* has also emerged as an etiological agent of gastroenteritis outbreaks in China and Japan, which are suspected to be transmitted through contaminated food and water (Asoshima et al., 2014; Masuda et al., 2020; Huang et al., 2024). *E. albertii* has been frequently misidentified as *Hafnia alvei*, *Shigella boydii*, and *Escherichia coli* (*E. coli*), leading to a severe underestimation of the clinical hazards (Hinenoya et al., 2019). This growing threat necessitates enhanced surveillance and comprehensive research to inform effective public health responses.

*E. albertii* has demonstrated a rapid progression in resistance, from previously susceptibility profiles to multidrug-resistant strains (defined here as resistance to three or more antimicrobial categories) (Magiorakos et al., 2012), including resistance to last-line agents such as carbapenems (Wang et al., 2022) and colistin (Li et al., 2018). The genomic evidence of a broad resistome aligns with this multidrug resistance (MDR) phenotype (Luo et al., 2021). Of particular concern is the detection of high-risk resistance determinants in some strains, including the extended-spectrum β-lactamase (ESBL) gene *bla*_CTX–M–55_ (Wang et al., 2022; Guo et al., 2024) and the mobile colistin resistance gene *mcr-1* (Li et al., 2018; Sonnevend et al., 2022), which collectively confer resistance to β-lactam/carbapenem and polymyxin antibiotics, respectively. These findings also underscore the potential for *E. albertii* to evolve multidrug resistance, driven by the acquisition of plasmids that accumulate horizontally transferred resistance mechanisms. However, current studies on MDR in *E. albertii* have primarily focused on strains from animal or food sources, leaving a significant gap in the characterization of clinically derived strains. The molecular mechanisms underlying resistance and the pathways by which it is acquired in clinical isolates warrant further investigation.

In our previous investigation of human-animal transmission (Zhang et al., 2025), we identified a multidrug-resistant *E. albertii* strain, ESA311, originally isolated from an infant presenting with diarrhea. It carries more than 20 antimicrobial resistance genes (ARGs), featuring a relatively complex antibiotic resistance profile. Critically, therapeutic failure was observed in the infected neonate following monotherapy with the third-generation cephalosporin cefotaxime, highlighting grave clinical challenges and underscoring the risk of treatment failure in pediatric patients. In addition, ESA311 harbors multiple plasmids simultaneously. Given the well-documented role of plasmids as mobile genetic elements that facilitate horizontal gene transfer of antimicrobial resistance determinants (Castañeda-Barba et al., 2024), their prevalence in ESA311 raises important questions regarding the mechanisms driving the spread of resistance in this pathogen. Our current study aims to perform a comprehensive genomic analysis of this clinical *E. albertii* strain, with particular emphasis on characterizing the resistance gene content and transfer potential of its plasmids, thereby elucidating their evolutionary history.

## 2 Materials and methods

### 2.1 Bacterial strains and growth conditions

The *E. albertii* strain ESA311, assigned to sequence type ST4606 and serotype EAOg1:EAHg4, was isolated from an infant suffering from diarrhea in Sichuan province, China (Zhang et al., 2025). *E. coli* EC600 (rifampin-resistant) was used as the recipient strain in conjugation experiments. Bacteria were routinely grown at 37°C on either solid or liquid Luria-Bertani (LB) medium (Beijing Land Bridge Technology Co., Ltd., Beijing, China).

### 2.2 Antimicrobial susceptibility testing

The antimicrobial susceptibility profile was evaluated using the NMIC/ID-801 Panels on the BD Phoenix™ 100 automated system (Becton Dickinson Company, Sparks, MD, United States). The panel included 21 antibiotics across 9 drug classes: phenicols (chloramphenicol), β-lactams (ampicillin, cefotaxime, ceftazidime, cefoxitin, aztreonam, ceftazidime-avibactam, ampicillin-sulbactam, ertapenem, meropenem, imipenem), tetracyclines (tetracycline, tigecycline), macrolides (azithromycin), quinolones (ciprofloxacin, nalidixic acid), aminoglycosides (amikacin, streptomycin), diaminopyrimidines and sulfonamides (trimethoprim-sulfamethoxazole), nitrofurans (nitrofurantoin), and polymyxins (colistin). *E. coli* ATCC 25922 was used for quality control. Antimicrobial susceptibility was interpreted according to the Clinical and Laboratory Standards Institute (CLSI M100 ED34:2024) for *Enterobacteriaceae* (CLSI, 2024).

### 2.3 Whole genome sequencing and assembly

Genomic DNA of the *E. albertii* strain ESA311 was extracted from an overnight fresh culture using the Wizard^®^ Genomic DNA purification kit (Promega, Madison, WI, United States), and subsequently sequenced on both the PacBio Sequel II and DNBSEQ platforms at the Beijing Genomics Institute (BGI, Shenzhen, China). Detailed sequencing and assembly procedures have been described in our previous study (Zhang et al., 2025). Coding sequences (CDSs) and pseudogenes were predicted using Prokka v1.14.6 (Seemann, 2014) and RAST (Accessed 20 December 2024),^1^ and further annotated through BLASTP and BLASTN searches against the UniProtKB/Swiss-Prot (Accessed 24 December 2024)^2^ and NCBI non-redundant (NR) database (Sayers et al., 2024).

### 2.4 Detection of ARGs, mobile genetic elements, and virulence genes

The presence of ARGs in the genome of ESA311 was determined using ABRicate 1.0.1^3^ with default parameters against the ResFinder database (Zankari et al., 2012). The insertion sequences (ISs) flanking the ARGs in the genome of strain ESA311 were identified using ISfinder (Siguier et al., 2006). The Integrons containing the integrase (*intI*) and cassettes encoding accessory genes were detected and analyzed using IntegronFinder 2.0 (Néron et al., 2022). Additionally, potential virulence factors were screened by querying the Virulence Factor Database (VFDB) under default settings (Chen et al., 2016).

### 2.5 Determination of plasmid replicon types in strain ESA311

The replicon types of the plasmids contained by strain ESA311 were determined using PlasmidFinder 2.1.^4^ The analysis was conducted employing the database “*Enterobacteriaceae*”. Unless otherwise specified, the analysis was performed with a minimum coverage of 60% and a minimum identity of 95%.

### 2.6 Conjugation experiments

Conjugation experiments were carried out using a filter mating method, with ESA311 as the donor strain and *E. coli* EC600 (rifampin-resistant) as the recipient strain (Yuan et al., 2021). Briefly, both strains were cultured in LB broth and incubated overnight at 37°C. For each conjugation assay, 200 μL of donor culture were mixed with 600 μL of recipient culture (v:v = 1:3). The mixture was vortexed thoroughly and 100 μL of the suspension was transferred onto a cellulose filter membrane (pore size, 0.22 μm) placed on a LB agar plate. Following incubation at 37 °C for 16–18 h, bacteria from the membrane were scraped off and resuspended in 1 mL of sterile saline solution. Serial dilutions of the resuspension were prepared and plated onto selective LB agar media. Transconjugants carrying plasmid pESA311_1 were selected on LB agar plates supplemented with 50 μg/mL chloramphenicol and 100 μg/mL rifampin, whereas those carrying pESA311_2 were selected using 2 μg/mL ciprofloxacin and 100 μg/mL rifampin. Transfer efficiency was calculated based on colony counts of transconjugants relative to recipient cells, performed in triplicate (Zhu et al., 2013). To confirm the presence of the respective plasmids in the transconjugants, eight randomly selected colonies from each selection plate were subjected to PCR analysis using plasmid-specific primers (Supplementary Table 1).

### 2.7 Phylogenetic analysis of the MDR plasmids

To construct phylogenetic trees, we identified plasmid sequences from NCBI that shared a minimum identity of 95% with pESA311_1 (≥ 80% coverage; *n* = 114) and pESA311_2 (≥ 60% coverage; *n* = 20), as detailed in Supplementary Tables 2, 3, respectively. QUAST v5.2.0 was used to assess the quality of the downloaded genomes (Mikheenko et al., 2018). A pairwise Mash distance matrix was calculated using Mash v2.3 (Ondov et al., 2016). This matrix was then used to infer a phylogenetic tree via the Neighbor-Joining (NJ) method as implemented in FastME v2.1.6.3 (Lefort et al., 2015).

### 2.8 Statistical analysis and data visualization

A chi-square test was conducted to evaluate the association between the presence of *intI1* and MDR. Statistical analysis was performed using IBM SPSS Statistics 21, with a *p* < 0.05 considered statistically significant.

Circular plasmid maps were generated using Proksee (Grant et al., 2023) and subsequently annotated for clarity. Comparisons of MDR regions and generation of linear genomic maps were conducted using Easyfig v2.2.5 (Sullivan et al., 2011). All schematic diagrams were assembled and finalized using vector graphics software (Inkscape v1.4 or Adobe Illustrator 2020). The butterfly diagram and chord diagram were generated using the online data visualization platform at https://www.cnsknowall.com/ (accessed on 12 March 2025).

### 2.9 Nucleotide sequence accession number

The nucleotide sequence of the ESA311 chromosome has been deposited in GenBank under the accession number CP157783. The complete sequences of plasmids pESA311_1 through pESA311_5 have been submitted to GenBank under accession numbers CP157784 to CP157788. The accession numbers for the other plasmids and strains used in this study are provided in Supplementary Tables 2–4, respectively.

## 3 Results

### 3.1 Antimicrobial susceptibility profile of strain ESA311

Initial antimicrobial susceptibility testing performed at the clinical microbiology laboratory using the Siemens LAB POR system indicated that strain ESA311 was susceptible to cefotaxime (MIC ≤ 2 μg/mL) and other third-generation cephalosporins (Zhang et al., 2025). However, therapeutic failure following cefotaxime monotherapy in the infected neonate prompted comprehensive reevaluation of its resistance profile. To resolve this discrepancy, confirmatory antimicrobial susceptibility testing was performed using the NMIC/ID-801 panels on the BD Phoenix™ 100 automated system. This advanced platform revealed several critical reclassifications in susceptibility results (Table 1). Notably, Retesting results revealed an elevated minimal inhibitory concentration (MIC) for cefotaxime (16 μg/mL), classifying the strain as resistant—contrasting with the initial clinical report of susceptibility (≤ 2 μg/mL)—and aligning with the observed clinical treatment failure.

**TABLE 1:** Antimicrobial susceptibility and ARGs of *E. albertii* strain ESA311.

Antimicrobial category	Antimicrobial agent	MIC (μg/mL)	Interpretation *	ARGs
Phenicols	Chloramphenicol	> 32	R	*cmlA1^a^, floR^a^*
Cephalosporins^†^	Cefotaxime	16	R^#^	*bla*_OXA–1_ *^a^, bla*_TEM–1B_ *^b^*
	Ceftazidime	8	I ^#^	
	Cefoxitin	16	I ^#^	
Penicillins^†^	Ampicillin	> 32	R	
Monocyclic β-lactam^†^	Aztreonam	> 16	R ^#^	
β-lactam/β-lactamase inhibitor complex^†^	Ceftazidime-avibactam	1/4	S	
	ampicillin-sulbactam	32/16	R	
Carbapenems^†^	Ertapenem	< = 0.25	S	−
	Meropenem	0.25	S	
	Imipenem	2	S	
Tetracyclines	Tetracycline	> 16	R	*tet*(A)*^ab^*
	Tigecycline	0.5	S	
Macrolides	Azithromycin	> 64	R	*mef*(B)*^a^, mph*(A)*^b^*
Quinolones	Ciprofloxacin	> 2	R	*mdf*(A)*, qnrS1*^*b*^
Aminoglycosides	Nalidixic acid	> 32	R	*aac(6′)- Ib-cr ^a^, aph(4)- Ia^a^, aac(3)- IVa^a^, aac(3)- IId^b^, aadA2^a^, aadA5^b^, ant(3”)- Ia ^a^*
	Amikacin	8	I^#^	
	Streptomycin	32	S	
Diaminopyrimidines and sulfonamide	Trimethoprim-sulfamethoxazole	> 8/152	R	*dfrA12 ^a^, dfrA17^b^, sul1 ^ab^, sul2^a^, sul3 ^a^*
Nitrofurans	Nitrofurantoin	< = 32	S	−
Polymyxins	Colistin	0.5	S	−

*R, resistant; I, intermediate; S, susceptible (as recommended by CLSI guidelines. ^†^ Belongs to the β-lactam class of antimicrobials, which includes penicillins, cephalosporins, carbapenems, monobactams, and related combination agents. ^#^Discrepancies in susceptibility results compared to the former results. ^a^ Genes encoded on pESA311_1. ^b^ Genes encoded on pESA311_2.

Importantly, strain ESA311 remained susceptible to carbapenems, with a meropenem MIC of 0.25 μg/mL, suggesting potential alternative therapeutic options. However, ESA311 exhibited resistance to multiple non-β-lactam antibiotics including chloramphenicol, tetracycline, azithromycin, ciprofloxacin, nalidixic acid, and trimethoprim-sulfamethoxazole. Notably, it retained susceptibility to certain aminoglycosides such as amikacin and streptomycin, highlighting the complexity of its resistance profile.

### 3.2 Coexistence of five plasmids in strain ESA311

Whole-genome sequencing (WGS) revealed that the genome of *E. albertii* strain ESA311 consists of a single chromosome of 4,795,532 bp and five plasmids. The average GC content of the chromosome is 49.8%. All five plasmids have circular closed DNA sequences, designated pESA311_1 to pESA311_5, with sizes ranging from 42,365 to 209,400 bp. Plasmid characteristics, including size, GC content, and replicon types, are summarized in Table 2.

**TABLE 2:** Key features of the five plasmids identified in *E. albertii* strain ESA311.

Plasmid ID	Size (bp)	GC content (%)	Number of predicted CDSs	Replicon types	Conjugation transfer capacity^2^
pESA311_1	209,400	47.4	276	IncHI2/IncHI2A	Y
pESA311_2	95,634	54.4	130	IncFII(pHN7A8)	Y
pESA311_3	89,218	50.0	121	IncI1-Iα	Y
pESA311_4	51,081	45.4	91	IncFIB (AP001918)/IncFII ^1^	Y
pESA311_5	42,365	45.1	54	IncP-1	Y

^1^>60% coverage and > 90% sequence identity. ^2^Y, Capable of conjugation transfer.

**FIGURE 1:**
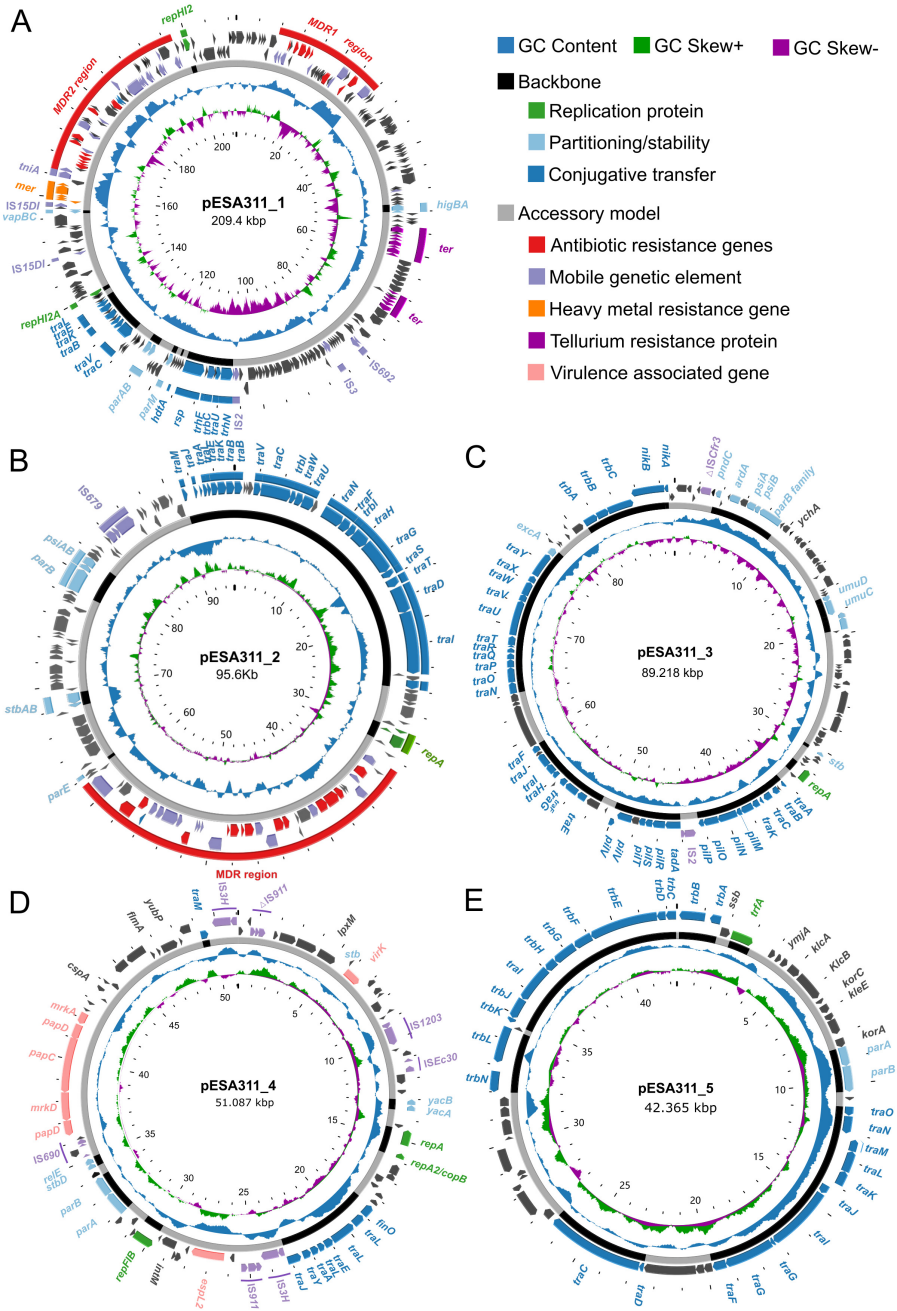
Schematic maps of the plasmids in ESA311. Genes are denoted by arrows and colored based on gene function classification. The innermost circle indicates the scale. The outer multi-peak circle illustrates GC skew [GC skew = (G−C)/(G+C)]. Purple inward peaks indicate genomic regions where guanine (G) content is lower than cytosine (C) (GC skew < 0). Green outward peaks denote regions with G > C (GC skew > 0). The next multi-peak shows G+C content (deviation from the average) in blue (outward peaks represent regions with G+C content higher than the average, inward troughs indicate G+C content below the average.). The black area in the next cycle represents the plasmid skeleton, while the gray area represents the accessory module regions. **(A)** pESA311_1; **(B)** pESA311_2; **(C)** pESA311_3; **(D)** pESA311_4; **(E)** pESA311_5.

Plasmid pESA311_1 (209,400 bp) contains 276 predicted CDSs and has an overall GC content of 47.4% (Figure 1A). It harbors two replicon types: IncHI2, showing 100% coverage and 100% sequence identity to the IncHI2 reference sequence of plasmid R478 (BX664015), and IncHI2A, which also exhibits 100% coverage but 99.52% sequence identity to the IncHI2A reference sequence of plasmid R478 (BX664015). Plasmid pESA311_2 (95,634 bp), containing 130 CDSs and a GC content of 54.4% (Figure 1B), was classified as an IncFII-type plasmid based on its alignment to the IncFII (pHN7A8) reference sequence (JN232517), exhibiting 100% coverage and 96.18% sequence identity to the replicon region. Similarly, pESA311_3 (89,218 bp, 121 CDSs, 50% GC content) (Figure 1C) displayed 100% coverage and 100% sequence identity when aligned to the IncI1α reference replicon sequence (AP005147). In contrast, pESA311_4 (51,081 bp, 91 CDSs, 45.4% GC content) (Figure 1D) could not be initially assigned to a specific replicon type. However, by lowering the identity threshold to 90%, we identified two replicon types—IncFIB (reference sequence AP001918) and IncFII (pSE11; reference sequence AP009242)—both with 100% coverage and identities of 93.84 and 93.94%, respectively. Finally, pESA311_5 (42,365 bp, 54 CDSs, 45.1% GC content) (Figure 1E) was identified as an IncP-1 plasmid based on its alignment to reference sequence CM007914.

Genomic annotation revealed distinct conjugative gene architectures among the five plasmids. pESA311_1 carries a set of *tra* genes (e.g., *traC*, *traV*, *traB*, *traK*, *traE*, *traL*). pESA311_2 possesses a complete IncF-type conjugative system, encompassing genes from *traA* to *traX*, including the relaxase gene *traI* and coupling protein gene *traD* (Figures 1A,B). pESA311_3 encodes a full IncI-type conjugative module with a comprehensive *tra*/*trb* cluster and the relaxase gene *nikB*. pESA311_4 harbors a truncated set of IncF-like *tra* genes (*traI*, *traA*, *traE*, *traL*, *traY*, *traJ*, *finO*). pESA311_5 contains key IncP-type transfer genes, including relaxase gene *traI*, coupling protein gene *traG*, and *trb* genes. Notably, pESA311_4 and pESA311_5 lack several core components typically found in autonomous conjugative systems, such as complete type four secretion system (T4SS) gene clusters for pilus assembly (Lawley et al., 2003; Fronzes et al., 2009).

### 3.3 ARGs and virulence-associated genes harbored by plasmids

Searches against the ResFinder database revealed the presence of multiple ARGs on plasmids pESA311_1 and pESA311_2 (Table 1). In contrast, only a single ARG, the major facilitator superfamily (MFS) efflux pump gene *mdf*(A), was identified on the chromosome of strain ESA311. This gene confers resistance to quinolones and contributes to MDR (Swick et al., 2011).

**FIGURE 2:**
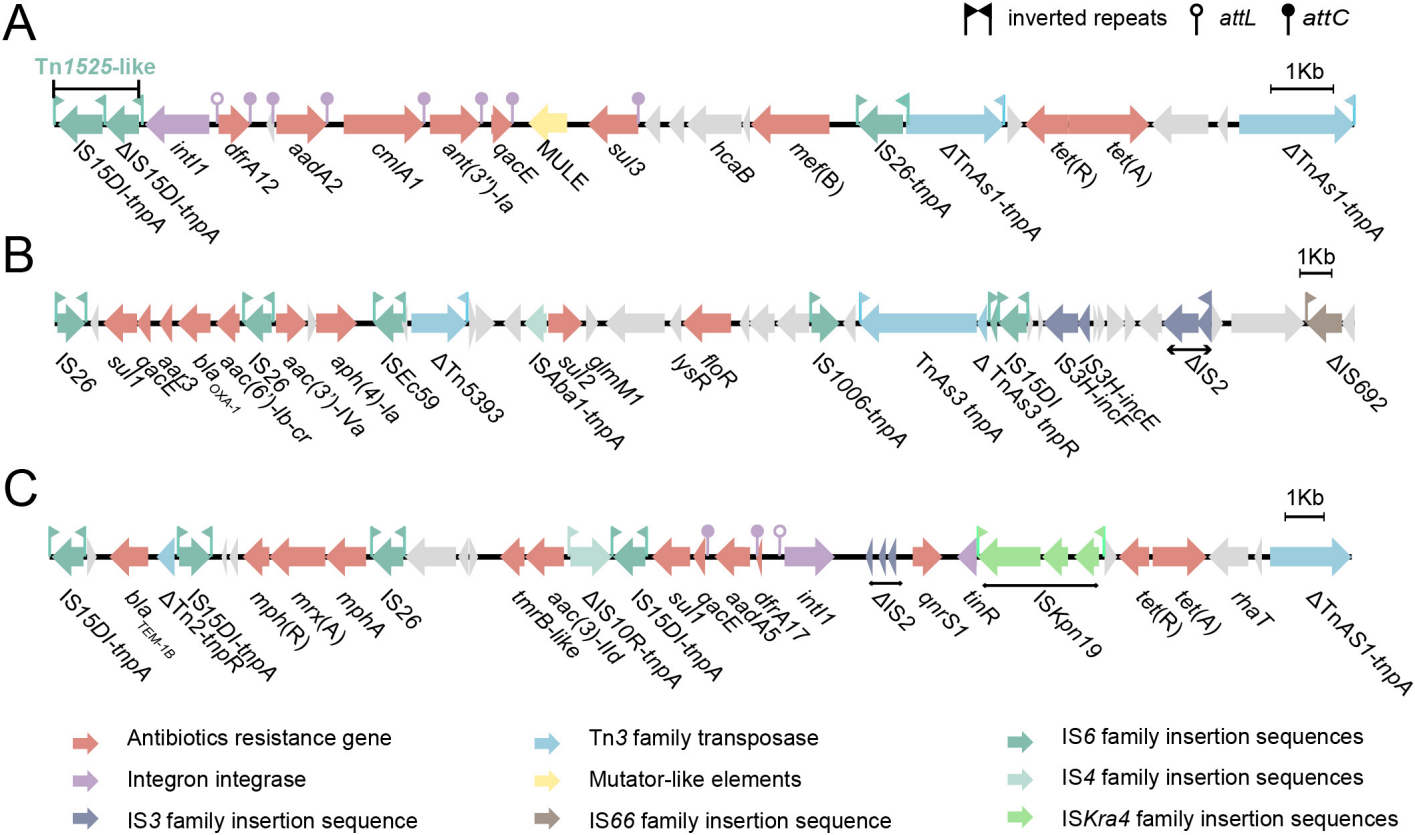
Structural organization of MDR regions in pESA311_1 and pESA311_2. Genes are denoted by arrows and colored based on gene function classification. **(A)** pESA311_1 MDR1 region; **(B)** pESA311_1 MDR2 region; **(C)** pESA311_2 MDR region.

The MDR plasmids pESA311_1 and pESA311_2 harbor a diverse repertoire of ARGs, conferring resistance to multiple classes of antibiotics, including phenicols (*cmlA, floR*), β-lactams (*bla*_OXA–1_*, bla*_TEM–1B_), tetracyclines [*tet*(A)], macrolides [*mef*(B), *mph*(A)], quinolones (*qnrS1*), aminoglycosides (*aac(6’)-Ib-cr*, *aph(4)-Ia*, *aac(3)- Ia*, *aac(3)- IId*, *aadA2*, *aadA5*, *ant(3”)- Ia*), sulfonamides (*sul1*, *sul2*, *sul3*), and diaminopyrimidines (*dfrA12*, *dfrA17*). The detailed visualization of the genetic organization and distribution of these ARGs were presented in Figures 1, 2, illustrating their clustering within specific regions of the plasmids. Such arrangements may facilitate co-selection and horizontal gene transfer.

The other three plasmids lacked detectable ARGs. However, pESA311_4 carried several virulence-associated genes, including *virK*, *espL2*, *papD*, *mrkD*, *papC*, and *mrkA* (Figure 1D). In contrast, pESA311_3 (Figure 1C) and pESA311_5 (Figure 1D) did not harbor any known virulence genes, highlighting the distinct genetic profiles of these plasmids. As shown in Figure 1, detailed annotation of the plasmids revealed differences in their genetic organization and functional potential, with pESA311_4 harboring a unique cluster of virulence genes that may contribute to its pathogenic potential.

### 3.4 Profiles of MDR regions on plasmids pESA311_1 and pESA311_2

To better understand the genetic organization of ARGs in plasmids pESA311_1 and pESA311_2, we analyzed the ARGs and their associated mobile genetic elements. The ARGs and the adjacent MGEs carried by pESA311_1 were clustered within two large MDR regions: a 20,362 bp fragment designated MDR1 and a 32,609 bp fragment designated MDR2 (Figure 1A).

The pESA311_1 MDR1 (Figure 2A) consists of two different transposable units. The first segment corresponds to a Tn*1525*-like transposon harboring a class I integron (*intI1*) cassette array [*intI1*-*dfrA12*-*aadA2*-*cmlA1*-*ant(3”)-Ia*-*qacE*-MULE (mutator-like element) —*sul3*] and a *mef*(B) gene. The second segment contains an incomplete Tn*AS1* remnant (ΔTn*AS1*), followed by the *tet*(R) and *tet*(A) genes, and ends with another ΔTn*AS1*. These two segments are connected by IS*26*.

The pESA311_1 MDR2 (Figure 2B) is flanked by IS*26* and ΔIS*692* and comprises a complex arrangement of mobile elements. It begins with an IS*26*-linked composite transposon containing *sul1*-*qacE*-*aar3*-*bla*_OXA–1_-*aac(6’)-Ib-cr*, followed by a second IS*26*-linked composite transposon harboring *aac(3’)-IVa-aph(4)-Ia-*IS*Ec59*-ΔTn*5393*-IS*Aba1*- *sul2*-*floR*-IS*1006*-ΔTn*AS3*. Additional ISs, including IS*15DI*, IS*3H*, ΔIS*2*, and ΔIS*692*, are also present in this region. Notably, mercury resistance-related genes embedded within an IS*15DI* element suggest that strain ESA311 may possess genetic determinants for mercury resistance (Figure 1A).

The ARGs and adjacent ISs carried by pESA311_2 were clustered within a 28,830 bp fragment (Figure 2C), which was organized sequentially as a β-lactam resistant gene, *bla*_TEM–1B_, flanked by IS*15DI* and ΔTn*2*, IS*15DI*-*mph*(R)-*mrxA*-*mph*(A)- IS*26*-*tmrB-like*-*aac(3)-IId* module, and a *intI1* cassette array (*intI1*-*dfrA17*-*aadA5*-*qacE*-*sul1*) carried by IS*15DI* on the negative strand, with further elements including ΔIS*2*, *qnrS1*, IS*Kpn19*, *tet*(R), *tet*(A), and ΔTn*AS1*.

### 3.5 Conjugation transfer of the plasmids

To evaluate the conjugative transfer potential of the antibiotic resistance plasmids pESA311_1 and pESA311_2, conjugation assays were performed using *E. coli* EC600, a rifampin-resistant strain, as the recipient. Transconjugants were selected on media containing chloramphenicol (50 μg/mL; selecting for pESA311_1) or ciprofloxacin (2 μg/mL; selecting for pESA311_2). The calculated conjugation frequencies were 5.30 × 10^−3^ and 4.90 × 10^−3^, respectively.

To confirm the presence of the plasmids in the transconjugants, eight randomly selected colonies from each antibiotic selection plate were subjected to PCR analysis using plasmid-specific primers (Supplementary Table 1). A striking pattern emerged: all transconjugants obtained under either selection regimen were found to carry a conserved set of plasmids—pESA311_1, pESA311_2, and pESA311_5 (Supplementary Figure 1). Notably, one ciprofloxacin-resistant transconjugant (Transconjugant 1) was found to additionally harbor pESA311_3, while another ciprofloxacin-resistant transconjugant (Transconjugant 7) carried pESA311_4. These observations highlight the potential for co-transfer of multiple plasmids, which may facilitate the spread of MDR among bacterial populations.

### 3.6 Phylogenetic analysis of the pESA311_1 and pESA311_2

**FIGURE 3:**
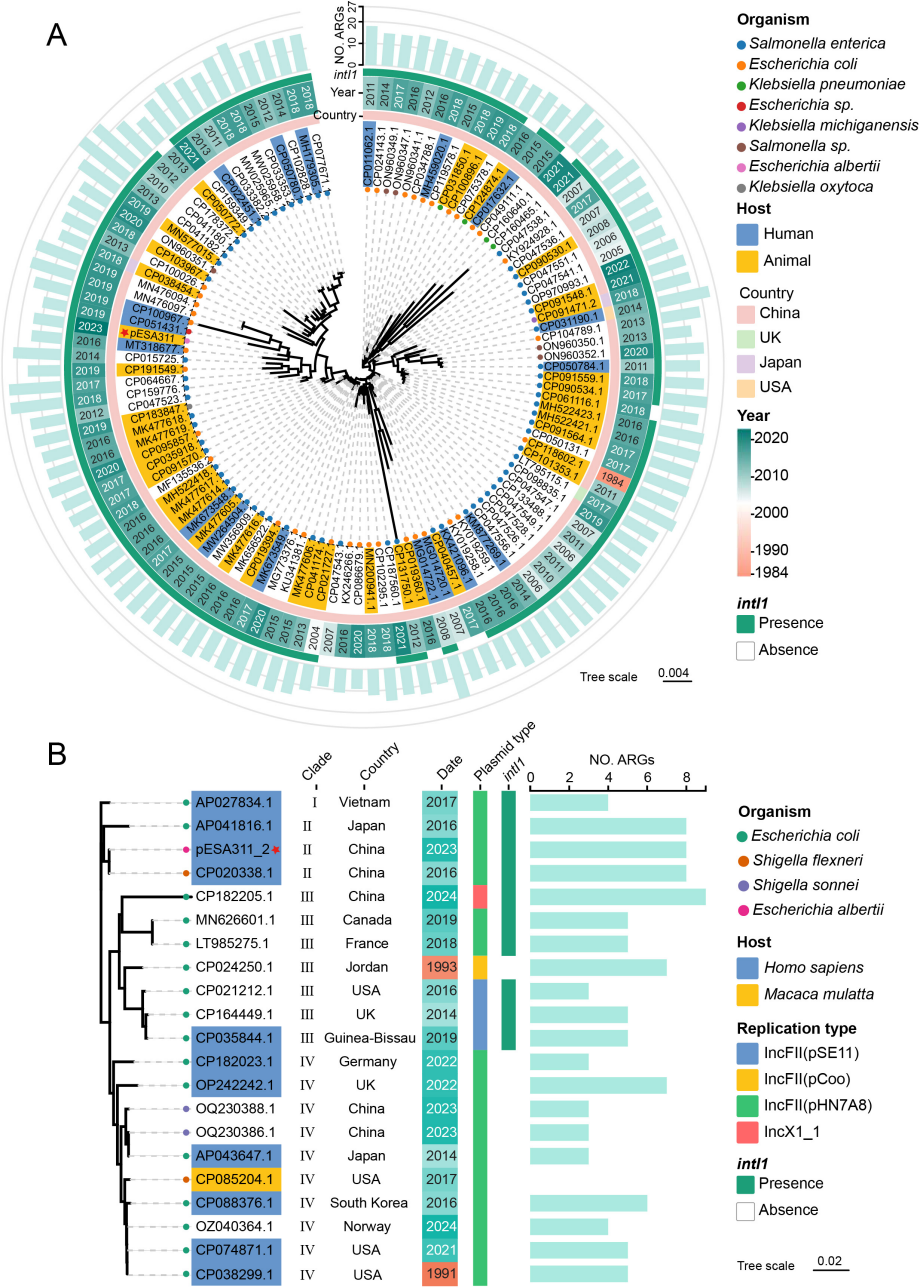
Phylogenetic analysis of pESA311_1 **(A)** and pESS311_2 **(B)**. The inclusion criteria for phylogenetic analysis were plasmid sequences with ≥ 95% identity to pESA311_1 (≥ 80% coverage; *n* = 114) or pESA311_2 (≥ 60% coverage; *n* = 20). The red star marks the plasmid used in this study. Organism: the bacterial species carrying the plasmid. Host: the source of the bacterial isolate.

The phylogenetic analysis revealed that the pESA311_1 related plasmid sequences were isolated from 1984 to 2023, with a significant increase in prevalence after 2018 (Supplementary Table 2 and Figure 3A). They were primarily hosted by *Salmonella enterica* and *E. coli* and exhibited a pronounced geographical clustering, with the overwhelming majority (96.5%) originating from China. Despite sharing the highest global sequence similarity with CP100967.1 (97.0% coverage, 100% identity), pESA311_1 was phylogenetically most closely related to plasmids MT318677.1 and CP051431.1, both of which were obtained from animal-derived strains. The plasmid phylogeny showed a high prevalence of the *intI1* integron (111, 89.5%), with ARGs counts ranging from 9 to 27 across these plasmids.

Despite a limited set of 21 homologous plasmids (>60% coverage, >95% identity) identified for pESA311_2, phylogenetic analysis revealed a broad geographical distribution across 12 countries and significant genetic diversity (Supplementary Table 3 and Figure 3B). While the predominant replicon type was IncFII(pHN7A8), matching pESA311_2, other types including IncFII(pSE11), IncFII(pCoo), and IncX1 were also detected. The phylogeny segregated the plasmids into four distinct Clades. Plasmid pESA311_2 resided in Clade II, forming a subclade with its closest relatives, AP041816.1 and CP020338.1. The *intI1* integron was universally present in Clades I, II, and III but was absent in Clade IV. Regarding host sources, most isolates with available data were of human origin, with a single strain isolated from the rhesus macaque (*Macaca mulatta*). Notably, this *Macaca mulatta*-derived strain lacked any detectable resistance genes, whereas all other strains carried between 3 and 9 resistance genes.

### 3.7 Prevalence of *intI1* in clinical *E. albertii* isolates

Two distinct *intI1*-borne gene cassette arrays form integral components of the MDR regions in strain ESA311, with one located on plasmid pESA311_1 and the other on pESA311_2. We analyzed whole-genome sequencing data from 160 clinical *E. albertii* isolates, comprising publicly available sequences and strains maintained by our research group (Supplementary Table 4). All isolates carried at least one ARG, and 22.5% (36/160) were predicted to be MDR (resistant to three or more tested antibiotic classes as defined by Magiorakos et al., 2012). Our analysis revealed that *intI1* was present in 20.0% (32/160) of the isolates.

**FIGURE 4:**
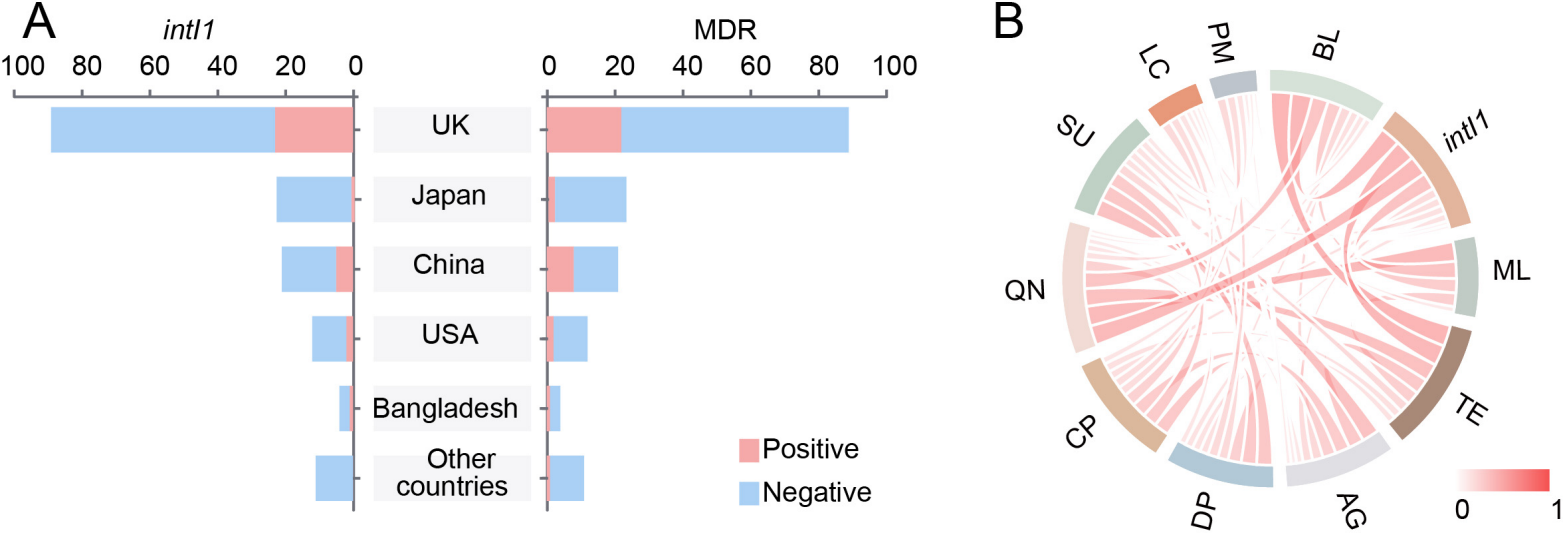
Distribution of *intI1* and its association with MDR in clinical *E. albertii* isolates. **(A)** Prevalence of *intI1*-positive and MDR *E. albertii* strains across different countries. **(B)** Chord diagrams visualizing the co-occurrence patterns between *intI1* and different classification of ARGs. BL, beta-lactams; ML, macrolides; TE, tetracyclines; AG, aminoglycosides; DP, diaminopyrimidines; CP, chloramphenicols; QN, quinolones; SU, sulfonamides; LC, lincosamides; PM, polymyxins.

Notably, the geographical distribution patterns of *intI1*-positive and multidrug-resistant strains were highly similar (Figure 4A). A strong positive correlation was observed between the presence of *intI1* and MDR status (φ = 0.816, *p* < 0.01). Furthermore, the Chord diagram (Figure 4B) revealed the frequent co-occurrence patterns between *intI1* and ARGs conferring resistance to β-lactam antibiotics, tetracycline antibiotics, macrolide antibiotics, and quinolone antibiotics.

## 4 Discussion

The antimicrobial resistance of *E. albertii* is increasingly recognized (Li et al., 2018; Wang et al., 2022); however, a key weakness in the field is the lack of comprehensive characterization of strains from clinical settings. The resistance profile of ESA311 highlights the growing challenge of multidrug-resistant *E. albertii* in clinical settings. Its resistance to third-generation cephalosporins, such as cefotaxime, coupled with susceptibility to carbapenems, such as ertapenem and meropenem, reflects the global trend of ESBL-producing *Enterobacteriaceae* (Subramanya et al., 2021; Sivarajan et al., 2025). However, strain ESA311 carried only the narrow-spectrum β-lactam resistance genes *bla*_OXA–1_ and *bla*_TEM–1B_. Sequencing confirmed that both alleles were unmutated, and no known genes conferring resistance to third-generation cephalosporins were detected. Thus, the resistance to cefotaxime in strain ESA311, may be attributed to a combination of mechanisms (Moya-Torres et al., 2014; Trampari et al., 2022). In particular, we noted that its *ompF* gene shares 88.72% identity with the *E. coli* ortholog (data not shown). Since functional OmpF porins are essential for the influx of cephalosporins, any sequence variation or expression defect in ESA311’s OmpF could significantly reduce the intracellular concentration of cefotaxime, thereby acting in concert with its chromosomal β-lactamases to confer clinical resistance.

Plasmids are key drivers of antibiotic resistance in Gram-negative bacteria (Abdallah et al., 2023; Chopjitt et al., 2024), and the five plasmids identified in strain ESA311 illustrate their diverse roles in both antimicrobial resistance and virulence. The MDR regions in plasmids pESA311_1 and pESA311_2 reveal complex genetic architectures shaped by diverse mobile elements, highlighting their role in the acquisition and dissemination of antibiotic resistance genes (Chen et al., 2022; Guan et al., 2022). The presence of multiple IS elements, transposons, and integrons underscores the dynamic evolutionary potential of these plasmids in adapting to antimicrobial selective pressures (Kotlarska et al., 2015; Gupta et al., 2022). Of particular significance are the *intI1*-mediated gene cassette arrays, including *dfrA12*, *aadA2*, and *cmlA1*, which are known to facilitate the acquisition, rearrangement, and expression of diverse resistance genes (Gillings, 2017; Puangseree et al., 2024). Furthermore, the presence of mercury resistance genes alongside ARGs on pESA311_1 suggests that environmental factors, such as heavy metal pollution, may contribute to the selection and persistence of MDR plasmids (Dickinson et al., 2019). Meanwhile, plasmid pESA311_4 carries several virulence-associated genes, including *virK*, *espL2*, *papD*, and *mrkD*, which are implicated in bacterial adhesion, invasion, and cytotoxic effects (Slonim et al., 1992; Tobe, 2010; Tapia-Pastrana et al., 2012; Stahlhut et al., 2013).

Our conjugation experiments revealed a striking and consistent transfer pattern: selection for either chloramphenicol (pESA311_1) or ciprofloxacin (pESA311_2) resistance invariably yielded transconjugants carrying a conserved trio of plasmids—pESA311_1, pESA311_2, and pESA311_5. This indicates that these plasmids formed a stable co-transfer consortium, with additional plasmids pESA311_3 and pESA311_4 being sporadically co-mobilized. This phenomenon underscores a significant risk for the simultaneous dissemination of multiple resistance determinants and virulence factors across bacterial populations. The genetic architecture of these plasmids provides a coherent explanation for this obligate co-transfer. pESA311_2 possesses a complete, canonical IncF-type conjugative system, endowing it with efficient self-transfer capability. In contrast, pESA311_1 harbors only a truncated set of IncF-like *tra* genes and does not possess the canonical VirB/VirD4-like T4SS typically associated with IncHI2 plasmid conjugation (Garcillán-Barcia et al., 2009), and pESA311_5 carries an incomplete T4SS gene cluster (Fronzes et al., 2009). Therefore, the most parsimonious interpretation of our data is that pESA311_2 acted as the primary conjugative driver, facilitating the highly efficient mobilization of pESA311_1 and pESA311_5. This aligns perfectly with the established paradigm of mobilizable plasmids relying on the conjugative machinery of a helper plasmid (Smillie et al., 2010). The consistent inclusion of pESA311_3 and pESA311_4 in some transconjugants further demonstrates the permissiveness of this helper system. This obligate “helper-satellite” dynamic has profound clinical and ecological implications. It creates a fixed genetic package wherein antibiotic selection pressure targeting any single component (e.g., chloramphenicol resistance on pESA311_1 or ciprofloxacin resistance on pESA311_2) inevitably drives the co-dissemination of the entire ensemble. Consequently, the spread of MDR (mediated by pESA311_1 and pESA311_2) becomes inextricably linked to the spread of virulence potential (carried by pESA311_4) and other accessory traits, exemplifying a powerful co-selection mechanism. This phenomenon highlights the potential for plasmids to act as vehicles for the dissemination of both resistance and virulence determinants, further complicating efforts in infection prevention and control (Liu et al., 2017; Zhang et al., 2022).

Phylogenetic analysis revealed two MDR plasmids with clearly distinct evolutionary and transmission patterns. The plasmid lineage represented by pESA311_1 has undergone noticeable clonal expansion since 2018, primarily circulating among *Salmonella enterica* and *E. coli* in China. The high prevalence of the *intI1* integron within this group is strongly associated with the accumulation of 9–27 resistance genes. Furthermore, phylogenetic reconstruction indicated that pESA311_1 is most closely related to plasmids of animal origin, supporting the occurrence of zoonotic transmission. In contrast, homologs of pESA311_2, though limited in number, displayed broader geographic distribution and greater genotypic diversity. Phylogenetically, they separated into two major lineages—one carrying *intI1* and the other lacking it—suggesting divergent evolutionary adaptations. It is also noteworthy that a strain isolated from *Macaca mulatta* carried no detectable resistance genes, standing in sharp contrast to human-derived strains (carrying 3–9 resistance genes). This pattern strongly implies that human antibiotic use is a major driver in the maintenance and spread of these resistance determinants.

The *intI1* integron cassette arrays represent a core component of the MDR regions in strain ESA311. To further investigate the role of *intI1* in the acquisition and dissemination of antibiotic resistance in *E. albertii*, we performed a genome-based analysis of 160 clinical strains. The overall prevalence of *intI1* was 20.0%, slightly lower than that observed in *E. coli* (22%) (Ahangarkani et al., 2015) and *Acinetobacter baumannii* (25.7%) (Akrami et al., 2017), and significantly lower than the rates reported in *Salmonella* spp. (36%) (Asgharpour et al., 2014), *Klebsiella* spp. (36.6%) (Sabbagh et al., 2021), and *Pseudomonas aeruginosa* (37–40%) (Moradian Kouchaksaraei et al., 2012; Sabbagh et al., 2021). Notably, the prevalence of *intI1*-carrying strains varied considerably across different countries. Among the four countries with larger strain collections, the *intI1* positivity rate was higher in isolates from the United Kingdom and China compared to those from the USA and Japan. Moreover, the distribution patterns of *intI1*-positive and multidrug-resistant strains are highly correlated (φ = 0.816, *p* < 0.01), indicating the strong correlation between the presence of *intI1* and MDR. Further analysis revealed the frequent co-occurrence patterns between *intI1* and ARGs related to β-lactams, tetracyclines, macrolides, and quinolones, consistent with findings from previous studies (Ramsamy et al., 2020).

Despite these insights, this study has limitations. As it focuses on a single strain, broader conclusions require validation in larger collections. Functional characterization of resistance and virulence genes, as well as their expression under varying conditions, remains an important area for future work. Moreover, the roles of plasmids pESA311_3 and pESA311_5—on which no ARGs or virulence genes were identified—warrant further investigation.

Together, our data underscore the role of conjugative plasmids in MDR for the emerging pathogen *E. albertii*. The two MDR plasmids in ESA311 followed different dissemination routes: pESA311_1 showed signs of recent clonal spread within China, potentially connected to animal reservoirs, whereas pESA311_2 exhibited broader geographic distribution and higher genetic diversity. Plasmid co-transfer, together with the modular organization of resistance regions (which include ISs and integrons), points to an effective mechanism for co-disseminating resistance and virulence traits. Furthermore, the strong association between the *intI1* integron and resistance to β-lactams, tetracyclines, macrolides, and quinolones highlights its importance as a genetic driver in clinical environments. Future work should focus on extending genomic surveillance to larger and more diverse strain collections, experimentally characterizing key resistance and virulence genes, and investigating how environmental and host factors influence the transfer of these mobile genetic elements.

## Data Availability

The datasets presented in this study can be found in online repositories. The names of the repository/repositories and accession number(s) can be found in this article/Supplementary material.

## Footnotes

1
https://rast.nmpdr.org/

2
http://web.expasy.org/docs/swiss-prot_guideline.html

3
https://github.com/tseemann/abricate

4
https://github.com/genomicepidemiology/plasmidfinder
