# Supplementary material for: Genomic insights into multidrug resistance in clinical Escherichia albertii: plasmid coexistence, intI1 prevalence, and interspecies dissemination risk
Source: Front Microbiol. 2026 Feb 24;17:1778797. doi: 10.3389/fmicb.2026.1778797 (PMC12971918; doi:10.3389/fmicb.2026.1778797)
Supplement: Supplementary file 1 [file Data_Sheet_1.docx]

Supplementary Material

**Table S1** PCR primers used in this study

| Primers | Sequence (5' to 3') | Length of Product (bp) | Purposes |
| --- | --- | --- | --- |
| IncHI2A_F | ACGACATCAAGATAACTGGT | 557 | Determine the presence of plasmid pESA311_1 |
| IncHI2A_R | TCCGATAGATTCAAGTTCCC |  |  |
| IncFII_F | CACACCTTCCTGCACTTATG | 261 | Determine the presence of plasmid pESA311_2 |
| IncFII_R | TGATCGTTTAAGGAATTTTGTGG |  |  |
| IncI1-I_F | GAAAGTCGGACGGCAGAAT | 134 | Determine the presence of plasmid pESA311_3 |
| IncI1-I_R | TCCGCCAAGTTCGTAAGAAA |  |  |
| pESA311_4_F | GGGCTGTGTTTCCACTATG | 416 | Determine the presence of plasmid pESA311_4 |
| pESA311_4_R | CCACGGGACTTAAGCTCT |  |  |
| IncP1_F | TGAAGGGCAGACAGAGAAGCA | 650 | Determine the presence of plasmid pESA311_5 |
| IncP1_R | TCCGAGCCACACATCATCTTGA |  |  |

**Table S2** Plasmid sequences homologous to pESA311_1 used for phylogenetic analysis (≥95% identity and ≥80% coverage)

| Accession number | Length | Organism | Strain | Coverage | Identity | Country^*^ | Host | Host species | Date^*^ | *intI1* | Number of ARGs |
| --- | --- | --- | --- | --- | --- | --- | --- | --- | --- | --- | --- |
| KM877269.1 | 249144 | *Salmonella enterica* | GDS147 | 88.0% | 99.9% | China | Chicken | Animal | 2014 | 1 | 18 |
| CP011062.1 | 255368 | *Escherichia coli* | Sanji | 85.0% | 100.0% | China | Pheasant | Animal | 2011 | 1 | 18 |
| KU341381.1 | 251493 | *Escherichia coli* | SHP45 | 87.0% | 100.0% | China | NA |  | 2015 | 1 | 16 |
| CP015725.1 | 210106 | *Salmonella enterica* | C629 | 82.0% | 100.0% | China | NA |  | 2014 | 1 | 22 |
| KX421096.1 | 253092 | *Salmonella enterica* | A3 | 85.0% | 100.0% | China | Chicken | Animal | 2016 | 1 | 15 |
| CP017632.1 | 369298 | *Escherichia coli* | SLK172 | 88.0% | 100.0% | China | Homo sapiens | Human | 2015 | 1 | 13 |
| KX246266.1 | 233814 | *Escherichia coli* | AHCH67 | 83.0% | 100.0% | China | NA |  | 2016 | 0 | 11 |
| KY019258.1 | 249152 | *Escherichia coli* | LDHF400 | 88.0% | 100.0% | China | NA |  | 2016 | 1 | 18 |
| KY019259.1 | 249153 | *Escherichia coli* | YJMC8 | 88.0% | 100.0% | China | NA |  | 2016 | 1 | 18 |
| KY924928.1 | 261119 | *Escherichia coli* | WJ1 | 87.0% | 100.0% | China | NA |  | 2017 | 1 | 18 |
| CP022451.1 | 222470 | *Salmonella enterica* | D90 | 85.0% | 100.0% | China | Chicken | Animal | 2013 | 1 | 21 |
| LT795115.1 | 246444 | *Salmonella enterica* | VNB151-sc-2315230 | 87.0% | 100.0% | UK | NA |  | 2017 | 1 | 17 |
| CP024143.1 | 254423 | *Escherichia coli* | 14EC029 | 84.0% | 100.0% | China | NA |  | 2014 | 1 | 15 |
| CP021727.1 | 224039 | *Escherichia coli* | Combat11I9 | 86.0% | 100.0% | China | Homo sapiens | Human | 2004 | 1 | 12 |
| MG014720.1 | 322966 | *Escherichia coli* | A74 | 83.0% | 100.0% | China | Duck | Animal | 2007 | 0 | 14 |
| MG014722.1 | 392275 | *Escherichia coli* | P2-3 | 91.0% | 100.0% | China | Pig | Animal | 2008 | 1 | 20 |
| MG773376.1 | 251657 | *Escherichia coli* | LSB54 | 87.0% | 100.0% | China | NA |  | 2020 | 1 | 16 |
| MH179305.1 | 244936 | *Salmonella enterica* | JT01 | 88.0% | 100.0% | China | Chicken | Animal | 2018 | 1 | 17 |
| MH459020.1 | 253783 | *Escherichia coli* | GZ6DS2 | 83.0% | 100.0% | China | Dog | Animal | 2018 | 1 | 14 |
| MF135536.2 | 250827 | *Escherichia coli* | HS20eCTX | 90.0% | 100.0% | China | NA |  | 2018 | 1 | 12 |
| CP034788.1 | 254365 | *Escherichia coli* | ECCNB20-2 | 83.0% | 100.0% | China | NA |  | 2016 | 1 | 14 |
| MK477605.1 | 252852 | *Salmonella enterica* | SH15G1428 | 87.0% | 100.0% | China | Homo sapiens | Human | 2015 | 1 | 16 |
| MK477606.1 | 254389 | *Salmonella enterica* | SH15G1450 | 87.0% | 100.0% | China | Homo sapiens | Human | 2015 | 1 | 16 |
| MK477614.1 | 251867 | *Salmonella enterica* | SH16G1394 | 87.0% | 100.0% | China | Homo sapiens | Human | 2016 | 1 | 15 |
| MK477616.1 | 251053 | *Salmonella enterica* | SH16G2456 | 87.0% | 100.0% | China | Homo sapiens | Human | 2016 | 1 | 15 |
| MK477617.1 | 249071 | *Salmonella enterica* | SH16G4466 | 87.0% | 100.0% | China | Homo sapiens | Human | 2016 | 1 | 16 |
| MK477618.1 | 219141 | *Salmonella enterica* | SH16G4511 | 86.0% | 100.0% | China | Homo sapiens | Human | 2016 | 1 | 14 |
| MK477619.1 | 217630 | *Salmonella enterica* | SH16G4918 | 87.0% | 100.0% | China | Homo sapiens | Human | 2016 | 1 | 14 |
| CP031850.1 | 239020 | *Klebsiella pneumoniae* | 121 | 80.0% | 100.0% | China | Homo sapiens | Human | 2018 | 1 | 13 |
| CP038454.1 | 211718 | *Escherichia coli* | EC-129 | 81.0% | 100.0% | Japan | Homo sapiens | Human | 2018 | 1 | 13 |
| CP033353.2 | 255327 | *Salmonella enterica* | CFSA664 | 90.0% | 100.0% | China | NA |  | 2015 | 1 | 23 |
| CP040457.1 | 254752 | *Salmonella enterica* | TJWQ005 | 84.0% | 100.0% | China | Homo sapiens | Human | 2017 | 0 | 14 |
| MH522418.1 | 244206 | *Salmonella enterica* | SH16G0648 | 87.0% | 100.0% | China | Homo sapiens | Human | 2016 | 1 | 14 |
| MH522421.1 | 299616 | *Salmonella enterica* | SH16G2457 | 90.0% | 100.0% | China | Homo sapiens | Human | 2016 | 1 | 21 |
| MH522423.1 | 297846 | *Salmonella enterica* | SH16G4498 | 90.0% | 100.0% | China | Homo sapiens | Human | 2016 | 1 | 21 |
| CP041174.1 | 248746 | *Salmonella enterica* | SJTUF12519v2 | 87.0% | 100.0% | China | Homo sapiens | Human | 2013 | 1 | 15 |
| CP041180.1 | 236217 | *Salmonella enterica* | SJTUF87912v2 | 85.0% | 100.0% | China | NA |  | 2010 | 1 | 26 |
| CP041182.1 | 240209 | *Salmonella enterica* | SJTUF13520v2 | 87.0% | 100.0% | China | NA |  | 2013 | 1 | 27 |
| MK673548.1 | 249627 | *Salmonella enterica* | GDP37-4 | 87.0% | 100.0% | China | Pig | Animal | 2017 | 1 | 16 |
| MK673549.1 | 244799 | *Salmonella enterica* | JXP9 | 84.0% | 100.0% | China | Pig | Animal | 2017 | 1 | 15 |
| CP035918.1 | 247705 | *Salmonella enterica* | S44712 | 88.0% | 100.0% | China | Homo sapiens | Human | 2017 | 1 | 13 |
| MN476094.1 | 320681 | *Escherichia coli* | NA | 96.0% | 100.0% | China | NA |  | 2019 | 1 | 21 |
| MN476097.1 | 296558 | *Escherichia coli* | NA | 96.0% | 100.0% | China | NA |  | 2019 | 1 | 21 |
| MN200941.1 | 241735 | *Escherichia coli* | E648 | 85.0% | 99.9% | China | Homo sapiens | Human | 2018 | 0 | 12 |
| MN577015.1 | 239910 | *Salmonella enterica* | NA | 84.0% | 100.0% | China | Homo sapiens | Human | 2019 | 1 | 21 |
| CP050772.1 | 199036 | *Salmonella enterica* | SI102 | 85.0% | 100.0% | China | Homo sapiens | Human | 2013 | 0 | 14 |
| CP050780.1 | 239058 | *Salmonella enterica* | SI85 | 90.0% | 100.0% | China | Chicken | Animal | 2012 | 1 | 16 |
| CP050784.1 | 255307 | *Salmonella enterica* | SI67 | 89.0% | 100.0% | China | Chicken | Animal | 2011 | 1 | 16 |
| CP051431.1 | 217533 | *Escherichia sp.* | SCLE84 | 90.0% | 100.0% | China | Swine | Animal | 2019 | 1 | 11 |
| MT318677.1 | 195614 | *Escherichia coli* | LWY24 | 87.0% | 100.0% | China | Chicken | Animal | 2016 | 1 | 14 |
| CP050131.1 | 298622 | *Salmonella enterica* | GSJ/2017-Sal-008 | 90.0% | 100.0% | China | NA |  | 2017 | 1 | 21 |
| CP033382.2 | 209798 | *Salmonella enterica* | CFSA300 | 83.0% | 100.0% | China | NA |  | 2011 | 1 | 18 |
| CP031190.1 | 308622 | *Salmonella enterica* | 222 | 90.0% | 100.0% | USA | Gallus | Animal | 2014 | 1 | 24 |
| CP064667.1 | 202724 | *Salmonella enterica* | SJTUF14154 | 82.0% | 100.0% | China | NA |  | 2017 | 1 | 24 |
| CP061116.1 | 284303 | *Salmonella enterica* | S585 | 86.0% | 100.0% | China | Homo sapiens | Human | 2018 | 0 | 17 |
| CP019360.1 | 349248 | *Escherichia coli* | XH993 | 83.0% | 100.0% | China | Homo sapiens | Human | 2016 | 0 | 11 |
| CP019394.1 | 248458 | *Escherichia coli* | XH992 | 86.0% | 100.0% | China | Homo sapiens | Human | 2016 | 1 | 14 |
| CP075378.1 | 279642 | *Escherichia coli* | no107 | 81.0% | 100.0% | China | NA |  | 2018 | 1 | 13 |
| MW264504.1 | 249043 | *Salmonella enterica* | S52 | 86.0% | 100.0% | China | Chicken | Animal | 2015 | 1 | 15 |
| CP077671.1 | 238101 | *Salmonella enterica* | S90 | 88.0% | 100.0% | China | NA |  | 2018 | 1 | 17 |
| CP047523.1 | 195873 | *Salmonella enterica* | SJTUF11216 | 88.0% | 100.0% | China | NA |  | 2012 | 1 | 16 |
| CP047526.1 | 249143 | *Salmonella enterica* | SJTUF11077 | 88.0% | 100.0% | China | NA |  | 2010 | 1 | 18 |
| CP047528.1 | 249152 | *Salmonella enterica* | SJTUF10648 | 88.0% | 100.0% | China | NA |  | 2011 | 1 | 18 |
| CP047536.1 | 243126 | *Salmonella enterica* | SJTUF10452 | 88.0% | 100.0% | China | NA |  | 2007 | 1 | 16 |
| CP047538.1 | 329730 | *Salmonella enterica* | SJTUF10405 | 87.0% | 100.0% | China | NA |  | 2007 | 1 | 15 |
| CP047541.1 | 232064 | *Salmonella enterica* | SJTUF10359 | 87.0% | 100.0% | China | NA |  | 2005 | 1 | 13 |
| CP047547.1 | 250477 | *Salmonella enterica* | SJTUF10236 | 88.0% | 100.0% | China | NA |  | 2007 | 1 | 18 |
| CP047549.1 | 249152 | *Salmonella enterica* | SJTUF10169 | 88.0% | 100.0% | China | NA |  | 2006 | 1 | 18 |
| CP047556.1 | 256080 | *Salmonella enterica* | SJTUF10112 | 88.0% | 100.0% | China | NA |  | 2006 | 1 | 18 |
| CP047551.1 | 240317 | *Salmonella enterica* | SJTUF10057 | 88.0% | 100.0% | China | NA |  | 2006 | 1 | 15 |
| CP047543.1 | 216197 | *Salmonella enterica* | SJTUF10330 | 83.0% | 100.0% | China | NA |  | 2007 | 0 | 9 |
| CP090530.1 | 241424 | *Salmonella enterica* | 2008079-SE | 88.0% | 100.0% | China | Homo sapiens | Human | 2008 | 1 | 15 |
| CP090534.1 | 292358 | *Salmonella enterica* | 2017005-SE | 88.0% | 100.0% | China | Homo sapiens | Human | 2017 | 1 | 20 |
| CP091471.2 | 286463 | *Klebsiella michiganensis* | KO_408 | 85.0% | 99.9% | Japan | Homo sapiens | Human | 2018 | 1 | 17 |
| MW025958.1 | 242443 | *Salmonella enterica* | 2-65 | 90.0% | 100.0% | China | NA |  | 2018 | 1 | 20 |
| MW356909.1 | 249475 | *Salmonella enterica* | S51 | 87.0% | 100.0% | China | NA |  | 2015 | 1 | 16 |
| MW025965.1 | 235653 | *Salmonella enterica* | 1-B7 | 90.0% | 100.0% | China | NA |  | 2018 | 1 | 16 |
| CP095857.1 | 230727 | *Escherichia coli* | EC6563 | 88.0% | 100.0% | China | Homo sapiens | Human | 2020 | 1 | 16 |
| CP091548.1 | 271705 | *Salmonella enterica* | 1559 | 88.0% | 100.0% | China | Homo sapiens | Human | 2021 | 1 | 17 |
| CP091559.1 | 287681 | *Salmonella enterica* | 418 | 88.0% | 100.0% | China | Homo sapiens | Human | 2018 | 1 | 18 |
| CP091564.1 | 295985 | *Salmonella enterica* | 143 | 90.0% | 100.0% | China | Homo sapiens | Human | 2017 | 1 | 20 |
| CP091570.1 | 247705 | *Salmonella enterica* | 131 | 88.0% | 100.0% | China | Homo sapiens | Human | 2017 | 1 | 13 |
| CP086679.1 | 262144 | *Escherichia coli* | EFF60 | 83.0% | 100.0% | China | NA |  | 2020 | 0 | 9 |
| CP098835.1 | 241147 | *Salmonella enterica* | GD19PS1 | 87.0% | 100.0% | China | NA |  | 2019 | 1 | 16 |
| CP100026.1 | 223975 | *Escherichia coli* | EC204A1 | 80.0% | 100.0% | China | NA |  | 2013 | 1 | 15 |
| CP102295.1 | 238291 | *Escherichia coli* | C1147 | 83.0% | 100.0% | China | Goose |  | 2018 | 0 | 15 |
| CP101353.1 | 263731 | *Salmonella enterica* | s11011 | 89.0% | 100.0% | China | Homo sapiens | Human | 2011 | 1 | 22 |
| CP102828.1 | 237848 | *Salmonella enterica* | XZ14C1328 | 90.0% | 100.0% | China | NA |  | 2014 | 1 | 18 |
| CP104789.1 | 259017 | *Escherichia coli* | SHP24 | 88.0% | 100.0% | China | NA |  | 2013 | 1 | 18 |
| ON960341.1 | 281462 | *Escherichia coli* | CBJ3C | 83.0% | 100.0% | China | NA |  | 2012 | 1 | 16 |
| ON960347.1 | 254841 | *Salmonella sp.* | 1.6E+243 | 84.0% | 100.0% | China | NA |  | 2016 | 1 | 16 |
| ON960349.1 | 257177 | *Salmonella sp.* | 17E557 | 84.0% | 100.0% | China | NA |  | 2017 | 1 | 15 |
| ON960350.1 | 242061 | *Salmonella sp.* | 13ESS0111 | 88.0% | 100.0% | China | NA |  | 2013 | 1 | 13 |
| ON960351.1 | 207851 | *Salmonella sp.* | a114 | 85.0% | 100.0% | China | NA |  | 2020 | 1 | 15 |
| ON960352.1 | 262175 | *Salmonella sp.* | quan12 | 89.0% | 100.0% | China | NA |  | 2020 | 1 | 18 |
| CP118602.1 | 261710 | *Escherichia coli* | NB4833 | 90.0% | 100.0% | China | Homo sapiens | Human | 1984 | 1 | 19 |
| CP119578.1 | 257019 | *Escherichia coli* | C012_chr | 83.0% | 100.0% | China | Broiler |  | 2015 | 1 | 14 |
| CP049111.1 | 436531 | *Escherichia coli* | WF5-5-T1 | 86.0% | 100.0% | China | NA |  | 2015 | 1 | 20 |
| CP124874.1 | 315303 | *Klebsiella pneumoniae* | K82 | 81.0% | 100.0% | China | Homo sapiens | Human | 2016 | 0 | 14 |
| MK656522.1 | 252976 | *Salmonella enterica* | Sa11291 | 87.0% | 100.0% | China | NA |  | 2016 | 1 | 16 |
| CP133488.1 | 249152 | *Salmonella enterica* | 94 | 88.0% | 100.0% | China | NA |  | 2011 | 1 | 18 |
| CP103967.1 | 216988 | *Salmonella enterica* | QT6365 | 84.0% | 100.0% | China | Homo sapiens | Human | 2018 | 1 | 16 |
| OP970993.1 | 253164 | *Salmonella enterica* | 6M-54 | 80.0% | 100.0% | China | Swine | Animal | 2022 | 1 | 16 |
| pESA311_1 | 209400 | *Escherichia albertii* | ESA311 | 100.0% | 100.0% | China | Homo sapiens | Human | 2023 | 1 | 14 |
| CP100896.1 | 273522 | *Escherichia coli* | ET846 | 83.0% | 100.0% | China | Hoplobatrachus | Animal | 2019 | 1 | 14 |
| CP100967.1 | 323994 | *Escherichia coli* | ET350 | 97.0% | 100.0% | China | Hoplobatrachus | Animal | 2019 | 1 | 17 |
| CP159349.1 | 214509 | *Salmonella enterica* | SJTUF15614 | 85.0% | 100.0% | China | NA |  | 2021 | 1 | 18 |
| CP178372.1 | 214046 | *Salmonella enterica* | IndS97 | 85.0% | 100.0% | China | Chicken |  | 2012 | 1 | 19 |
| CP160465.1 | 485030 | *Klebsiella pneumoniae* | J21CTR26 | 86.0% | 100.0% | China | Chicken |  | 2021 | 1 | 13 |
| CP160640.1 | 394261 | *Klebsiella pneumoniae* | J21CTR30 | 87.0% | 100.0% | China | Chicken |  | 2021 | 0 | 18 |
| CP183847.1 | 207701 | *Klebsiella oxytoca* | K1131 | 89.0% | 100.0% | China | Homo sapiens | Human | 2019 | 1 | 16 |
| CP131750.1 | 286967 | *Salmonella enterica* | 2012C07-028 | 82.0% | 97.7% | China | Homo sapiens | Human | 2012 | 1 | 14 |
| CP187560.1 | 250469 | *Salmonella enterica* | SJTUF15758 | 88.0% | 100.0% | China | NA |  | 2021 | 1 | 13 |
| CP191549.1 | 186992 | *Escherichia coli* | 19451123 | 81.0% | 100.0% | China | Homo sapiens | Human | 2019 | 1 | 15 |
| CP159776.1 | 216886 | *Salmonella enterica* | 18S246 | 86.0% | 100.0% | China | NA |  | 2018 | 1 | 24 |

^*^ For isolates with missing metadata, the submitter's country and sequence submission date (in red) were used as substitutes for the collection site and date, respectively.

Table S3 Plasmid sequences homologous to pESA311_2 used for phylogenetic analysis (≥95% identity and ≥60% coverage)

| Accession number | Length | Organism | Strain | Coverage | Identity | Country^*^ | Host | Host species | Date^*^ | *intI1* | Number of ARGs | Plasmid type |
| --- | --- | --- | --- | --- | --- | --- | --- | --- | --- | --- | --- | --- |
| CP021212.1 | 90229 | *Escherichia coli* | B171 | 62.0% | 99.4% | USA |  |  | 2016 | 1 | 3 | IncFII(pSE11) |
| CP024250.1 | 99099 | *Escherichia coli* | D181 | 61.0% | 98.2% | Jordan |  |  | 1993 |  | 7 | IncFII(pCoo) |
| CP020338.1 | 95633 | *Shigella flexneri* | 1602 | 100.0% | 100.0% | China | Homo sapiens | Human | 2016 | 1 | 8 | IncFII(pHN7A8) |
| LT985275.1 | 91487 | *Escherichia coli* | R71 | 65.0% | 98.2% | France |  |  | 2018 | 1 | 5 | IncFII(pHN7A8) |
| CP038299.1 | 70894 | *Escherichia coli* | TB182A | 64.0% | 98.9% | USA | Homo sapiens | Human | 1991 |  | 5 | IncFII(pHN7A8) |
| MN626601.1 | 91490 | *Escherichia coli* | BM21 | 65.0% | 98.2% | Canada |  |  | 2019 | 1 | 5 | IncFII(pHN7A8) |
| CP035844.1 | 93485 | *Escherichia coli* | TW10722 | 61.0% | 99.4% | Guinea-Bissau | Homo sapiens | Human | 2019 | 1 | 5 | IncFII(pSE11) |
| CP088376.1 | 71927 | *Escherichia coli* | F16EC0617 | 65.0% | 98.9% | South Korea | Homo sapiens | Human | 2016 |  | 6 | IncFII(pHN7A8) |
| CP085204.1 | 58626 | *Shigella flexneri* | P099 | 60.0% | 99.0% | USA | Macaca mulatta | Animal | 2017 |  | 0 | IncFII(pHN7A8) |
| OP242242.1 | 78662 | *Escherichia coli* | LA251 | 67.0% | 99.3% | UK | Homo sapiens | Human | 2022 |  | 7 | IncFII(pHN7A8) |
| OQ230386.1 | 76038 | *Shigella sonnei* | RY185 | 68.0% | 99.4% | China |  |  | 2023 |  | 3 | IncFII(pHN7A8) |
| OQ230388.1 | 77935 | *Shigella sonnei* | SH12sh288 | 72.0% | 99.1% | China |  |  | 2023 |  | 3 | IncFII(pHN7A8) |
| AP027834.1 | 80254 | *Escherichia coli* | 2017.17.03CC | 79.0% | 100.0% | Viet Nam | Homo sapiens | Human | 2017 | 1 | 4 | IncFII(pHN7A8) |
| pESA311_2 | 95634 | *Escherichia albertii* | ESA311 | 100.0% | 100.0% | China | Homo sapiens | Human | 2023 | 1 | 8 | IncFII(pHN7A8) |
| CP164449.1 | 88868 | *Escherichia coli* | OXEC-157 | 61.0% | 99.4% | UK |  |  | 2014 | 1 | 5 | IncFII(pSE11) |
| OZ040364.1 | 68914 | *Escherichia coli* | 30859_5 | 64.0% | 98.9% | Norway |  |  | 2024 |  | 4 | IncFII(pHN7A8) |
| CP074871.1 | 70888 | *Escherichia coli* | 2013C_3277 | 64.0% | 99.4% | USA | Homo sapiens | Human | 2021 |  | 5 | IncFII(pHN7A8) |
| CP182023.1 | 75264 | *Escherichia coli* | A28067 | 70.0% | 99.5% | Germany | Homo sapiens | Human | 2022 |  | 3 | IncFII(pHN7A8) |
| CP182205.1 | 106201 | *Escherichia coli* | JB2 | 64.0% | 95.2% | China |  |  | 2024 | 1 | 9 | IncX1_1 |
| AP041816.1 | 163363 | *Escherichia coli* | JNE21-009 | 93.0% | 100.0% | Japan | Homo sapiens | Human | 2016 | 1 | 8 | IncFII(pHN7A8) |
| AP043647.1 | 76047 | *Escherichia coli* | AST85 | 68.0% | 99.4% | Japan | Homo sapiens | Human | 2014 |  | 3 | IncFII(pHN7A8) |

* For isolates with missing metadata, the submitter's country and sequence submission date (in red) were used as substitutes for the collection site and date, respectively.

Table S4. Prevalence of *IntI1* and ARGs in clinical *E. albertii* isolates

| Accession number | Strain name | Region | Year | *intI1* | MDR types¹ | MDR^2^ | Antimicrobial resistance genes |
| --- | --- | --- | --- | --- | --- | --- | --- |
| GCA_022833075.1 | strain: NBRC 107761=EaTS | Bangladesh | 1990 |  |  | 0 |  |
| GCA_000208425.2 | strain: TW08933 | Bangladesh | 2003/3/13 |  |  | 0 |  |
| GCA_000512125.1 | strain: KF1 | Poland | 2013 |  |  | 0 |  |
| GCA_001514555.1 | strain: 20H38 | Japan | 2008 |  |  | 0 |  |
| GCA_001514575.1 | strain: 24 | Japan | 2000 |  | 3 | 1 | *tet*(A)*, aph(6)-Ⅰd_1, aph(3'')- Ⅰb_5, sul2* |
| GCA_001514595.1 | strain: Jun-51 | Brazil | 1989 |  |  | 0 |  |
| GCA_001514625.1 | strain: 94389 | Japan | 1994 |  |  | 0 |  |
| GCA_001514845.1 | strain: CB9791 | Germany | 2003 |  |  | 0 |  |
| GCA_001514885.1 | strain: EC03-127 | Japan | 2003 |  |  | 0 |  |
| GCA_001514905.1 | strain: EC03-195 | Japan | 2003 |  |  | 0 |  |
| GCA_001514925.1 | strain: EC05-160 | Japan | 2005 |  |  | 0 |  |
| GCA_001514945.1 | strain: EC05-44 | Japan | 2005 |  |  | 0 |  |
| GCA_001514965.1 | strain: EC05-81 | Japan | 2005 |  |  | 0 |  |
| GCA_001514985.1 | strain: HIPH08472 | Japan | 2008 |  |  | 0 |  |
| GCA_001515005.1 | strain: K7394 | Japan | 2008 |  |  | 0 |  |
| GCA_001515025.1 | strain: K7744 | Japan | 2009 |  |  | 0 |  |
| GCA_001515045.1 | strain: K7756 | Japan | 2009 |  |  | 0 |  |
| GCA_001515065.1 | strain: KU20110014 | Japan | 2011 |  | 0 |  |  |
| GCA_001549955.1 | strain: EC06-170 | Japan | 2006 |  |  | 0 |  |
| GCA_002285475.1 | strain: CB9786 | Germany | 2003 |  |  | 0 |  |
| GCA_002741375.1 | strain: 2014C-4356 | USA | 2014 | 1 | 3 | 1 | *tet*(A)*, aph(6)-Ⅰd_1, aph(3'')-Ib-2, aac(3)-Ⅵa-2, ant(3'')-Ia-1, sul1* |
| GCA_002895205.1 | strain: 1551-2 | Brazil | 1989 |  |  | 0 |  |
| GCA_002965635.1 | strain: MOD1-EC1733 | Guinea | 1997 |  |  | 0 |  |
| GCA_002965665.1 | strain: MOD1-EC1724 | USA | 2005 |  |  | 0 |  |
| GCA_002965685.1 | strain: MOD1-EC1732 | Guinea | 1997 |  |  | 0 |  |
| GCA_003312485.2 | strain: 05-3106 | USA | - | 1 | 5 | 1 | *bla*_TEM-1B_*, tet*(A)*, ant(3'')-Ia_1, dfrA15_2, sul1* |
| GCA_003312525.2 | strain: 2012EL-1823B | USA | 2012 |  |  | 0 |  |
| GCA_003312545.2 | strain: 2013C-4143 | USA | 2013 |  |  | 0 |  |
| GCA_003316815.2 | strain: 07-3866 | USA | - |  |  | 0 |  |
| GCA_003569025.1 | strain: Hiroshima3582 | Japan | 2015 |  |  | 0 |  |
| GCA_003860365.1 | strain: 06-3542 | USA | - |  |  | 0 |  |
| GCA_003860385.1 | strain: 2014C-4015 | USA | 2014 |  |  | 0 |  |
| GCA_003864075.1 | strain: 2010C-3449 | USA | 2010 |  | 2 | 0 | *bla*_TEM-1B_*, tet*(A) |
| GCA_003864095.1 | strain: NCTC 9362 | USA | 1954 |  |  | 0 |  |
| GCA_004174315.1 | strain: 408285 | UK | 2017/8/1 |  |  | 0 |  |
| GCA_004322685.1 | strain: Mex-12/320a | Mexico | 2012 |  | 5 | 1 | *bla*_TEM-1B_*, tet*(B)*_2, aph(6)-Id_1, aph(3'')-Ib_5, dfrA8_1, sul2* |
| GCA_005404525.1 | strain: Mkr3964 | Japan | - | 1 | 8 | 1 | *floR_2, bla*_TEM-135_1_*, tet*(A)*, tet*(M)*_4, qnrS1, aph(3')-Ia_3, aph(4)-Ia_1, aac(3)-Ⅳa_1, ant(3'')-Ia_1, dfrA5_1, sul1, sul3, mcr-1.1_1* |
| GCA_005404625.1 | strain: Mkr3965 | Japan | - |  |  | 0 |  |
| GCA_005404705.1 | strain: Esc18014 | Japan | - |  |  | 0 |  |
| GCA_008326565.1 | strain: MKR5 | Japan | 2012 |  | 2 | 0 | *tet*(B)*_2, aph(6)-Id_1, aph(3'')-Ib_2* |
| GCA_009684735.2 | strain: F08/101-31 | Japan | 2008 |  |  | 0 |  |
| GCA_009932235.1 | strain: FCI-EC447 | Japan | 2003 |  |  | 0 |  |
| GCA_009932275.1 | strain: FCI-EC468 | Japan | 2005 |  |  | 0 |  |
| GCA_009932295.1 | strain: 13S38 | Japan | 2013 |  |  | 0 |  |
| GCA_012097705.1 | strain: 579962 | UK | 2018/7/1 |  |  | 0 |  |
| GCA_012117435.1 | strain: 660450 | UK | 2018/12/1 |  | 1 | 0 | *bla*_TEM-106_1_ |
| GCA_012161415.1 | isolate: 2011C-4180 | USA | - |  |  | 0 |  |
| GCA_012163415.1 | strain: 664948 | UK | 2018/12/1 | 1 | 3 | 1 | *bla*_TEM-1B_1_*, tet*(A)*, aph(6)-Id_1, aph(3'')-Ib_5* |
| GCA_012287085.1 | strain: 762368 | UK | 2018/6/1 | 1 | 4 | 1 | *bla*_TEM-1B_1_*, bla*_DHA-1_1_*, tet*(A)*, mph*(A)*_2, qnrB4_1* |
| GCA_012288225.1 | strain: 762329 | UK | 2019/6/1 | 1 | 4 | 1 | *bla*_TEM-1B_1_*, bla*_DHA-1_1_*, tet*(A)*, mph*(A)*_2, qnrB4_1* |
| GCA_012292265.1 | strain: 730730 | UK | 2019/4/1 |  |  | 0 |  |
| GCA_012303345.1 | strain: 779119 | UK | 2019/7/1 | 1 | 4 | 1 | *bla*_TEM-1B_1_*, bla*_DHA-1_1_*, tet*(A)*, mph*(A)*_2, qnrB4_1* |
| GCA_012319645.1 | strain: 709086 | UK | 2019/3/1 | 1 | 3 | 1 | *bla*_TEM-1B_1_*, tet*(A)*, qnrB19_1* |
| GCA_012330025.1 | strain: 727738 | UK | 2019/3/2 | 1 | 4 | 1 | *bla*_TEM-1B_1_*, bla*_DHA-1_1_*, tet*(A)*, mph*(A)*_2, qnrB4_1* |
| GCA_012331125.1 | strain: 712432 | UK | 2019/3/3 | 1 | 3 | 1 | *bla*_TEM-1B_1_*, tet*(A)*, qnrB19_1* |
| GCA_012336555.1 | strain: 782039 | UK | 2019/7/1 |  |  | 0 |  |
| GCA_012338195.1 | strain: 730926 | UK | 2019/2/1 |  |  | 0 |  |
| GCA_012340675.1 | strain: 714709 | UK | 2019/3/3 | 1 | 4 | 1 | *bla*_TEM-1B_1_*, bla*_DHA-1_1_*, tet*(A)*, mph*(A)*_2, qnrB4_1* |
| GCA_012376075.1 | strain: 322491 | UK | 2016/12/1 |  |  | 0 |  |
| GCA_012379895.1 | strain: 738553 | UK | 2019/4/1 | 1 | 4 | 1 | *bla*_TEM-1B_1_*, bla*_DHA-1_1_*, tet*(A)*, mph*(A)*_2, qnrB4_1* |
| GCA_012401465.1 | strain: 785841 | UK | 2019/7/1 | 1 | 4 | 1 | *bla*_TEM-1B_1_*, bla*_DHA-1_1_*, tet*(A)*, mph*(A)*_2, qnrB4_1* |
| GCA_012447505.1 | strain: 821093 | UK | 2019/10/1 | 1 | 2 | 0 | *bla*_TEM-1B_1_*, tet*(A) |
| GCA_012532395.1 | strain: E. albertii 10457 | Bangladesh | 1990 |  | 3 | 1 | *bla*_TEM-1B_1_*, tet*(B)*_2, aph(6)-Id_1, aph(3'')-Ib_5* |
| GCA_012532475.1 | strain: E. albertii 9194 | Bangladesh | 1990 | 1 | 2 | 0 | *bla*_TEM-1B_1_*, tet*(B)*_2* |
| GCA_012565605.1 | strain: 267803 | UK | 2016/6/1 |  |  | 0 |  |
| GCA_012575105.1 | strain: 290626 | UK | 2016/8/1 |  | 1 | 0 | *bla*_TEM-1B_1_ |
| GCA_012575625.1 | strain: 175546 | UK | 2015/10/1 |  |  | 0 |  |
| GCA_012579975.1 | strain: 253881 | UK | 2016/5/1 | 1 | 3 | 1 | *bla*_TEM-1B_1_*, tet*(A)*, aph(6)-Id_1, aph(3'')-Ib_2* |
| GCA_012612125.1 | strain: 841428 | UK | 2019/11/1 | 1 | 4 | 1 | *bla*_TEM-1B_1_*, blaDHA-1_1, tet*(A)*, mph*(A)*_2, qnrB4_1* |
| GCA_012622165.1 | strain: 828215 | UK | 2019/10/1 | 1 | 2 | 0 | *bla*_TEM-1B_1_*, tet*(A) |
| GCA_012622265.1 | strain: 826231 | UK | 2019/10/2 |  |  | 0 |  |
| GCA_012625565.1 | strain: 812315 | UK | 2019/9/1 |  |  | 0 |  |
| GCA_012678705.1 | strain: 187471 | UK | 2015/10/1 |  | 2 | 0 | *bla*_TEM-1B_1_*, tet*(A) |
| GCA_012678765.1 | strain: 159899 | UK | 2015/9/1 |  |  | 0 |  |
| GCA_012679445.1 | strain: 186581 | UK | 2015/11/1 |  | 1 | 0 | *bla*_TEM-1B_1_ |
| GCA_012679585.1 | strain: 242400 | UK | 2016/4/1 |  |  | 0 |  |
| GCA_012679905.1 | strain: 225083 | UK | 2016/2/1 | 1 | 4 | 1 | *bla*_TEM-1B_1_*, bla*_DHA-1_1_*, tet*(A)*, mph*(A)*_2, qnrB4_1* |
| GCA_012681005.1 | strain: 211128 | UK | 2016/1/1 | 1 | 3 | 1 | *bla*_TEM-1B_1_*, tet*(A)*, aph(6)-Id_1, aph(3'')-Ib_5* |
| GCA_012681465.1 | strain: 235021 | UK | 2016/3/1 |  |  | 0 |  |
| GCA_012690285.1 | strain: 884403 | UK | 2020/1/1 | 1 | 4 | 1 | *bla*_TEM-1B_1_*, bla*_DHA-1_1_*, tet*(A)*, mph*(A)*_2, qnrB4_1* |
| GCA_012850675.1 | strain: 914519 | UK | 2020/3/1 |  |  | 0 |  |
| GCA_012854695.1 | strain: 912319 | UK | 2020/3/2 |  |  | 0 |  |
| GCA_012854805.1 | strain: 912272 | UK | 2020/3/3 | 1 | 3 | 1 | *bla*_TEM-1B_1_*, tet*(A)*, aph(6)-Id_1, aph(3'')-Ib_5* |
| GCA_012996545.1 | strain: 787519 | UK | 2019/7/1 |  |  | 0 |  |
| GCA_013580255.1 | strain: 961868 | UK | 2020/7/1 |  |  | 0 |  |
| GCA_013680375.1 | strain: 953783 | UK | 2020/6/1 |  |  | 0 |  |
| GCA_014355615.1 | strain: 977893 | UK | 2020/8/1 |  |  | 0 |  |
| GCA_014402965.1 | strain: 419115 | UK | 2020/9/2 |  |  | 0 |  |
| GCA_014404825.1 | strain: 416278 | UK | 2020/9/3 |  | 1 | 0 | *tet*(B)*_2* |
| GCA_014404845.1 | strain: 435509 | UK | 2017/10/1 |  |  | 0 |  |
| GCA_014408585.1 | strain: 323046 | UK | 2016/12/1 |  | 3 | 1 | *bla*_TEM-1B_1_*, tet*(B)*_2, aph(6)-Id_1, aph(3'')-Ib_5* |
| GCA_014413465.1 | strain: 385522 | UK | 2017/6/1 |  |  | 0 |  |
| GCA_014415845.1 | strain: 367208 | UK | 2017/4/1 |  |  | 0 |  |
| GCA_014417525.1 | strain: 378483 | UK | 2017/6/1 |  |  | 0 |  |
| GCA_014429205.1 | strain: 379422 | UK | 2017/6/2 |  |  | 0 |  |
| GCA_014429445.1 | strain: 416277 | UK | 2017/9/1 |  |  | 0 |  |
| GCA_015135085.1 | strain: 802832 | UK | 2019/9/1 | 1 | 4 | 1 | *bla*_TEM-1B_1_*, bla*_DHA-1_1_*, tet*(A)*, mph*(A)*_2, qnrB4_1* |
| GCA_015135955.1 | strain: 882856 | UK | 2020/1/1 |  |  | 0 |  |
| GCA_015135975.1 | strain: 810189 | UK | 2019/9/1 |  |  | 0 |  |
| GCA_015136275.1 | strain: 808053 | UK | 2019/9/2 |  |  | 0 |  |
| GCA_015137055.1 | strain: 819793 | UK | 2019/10/4 |  |  | 0 |  |
| GCA_015141875.1 | strain: 810124 | UK | 2019/9/2 |  |  | 0 |  |
| GCA_016998775.1 | isolate: M876 | Netherlands | 2011 |  |  | 0 |  |
| GCA_016999345.1 | isolate: 1701358 | Netherlands | 2017 |  |  | 0 |  |
| GCA_017953935.1 | strain: 1049314 | UK | 2020/12/1 |  |  | 0 |  |
| GCA_019470745.1 | strain: 716041 | UK | 2019/3/1 | 1 | 4 | 1 | *bla*_TEM-1B_1_*, bla*_DHA-1_1_*, tet*(A)*, mph*(A)*_2, qnrB4_1* |
| GCA_019478965.1 | strain: 832087 | UK | 2019/10/1 |  |  | 0 |  |
| GCA_019480245.1 | strain: 786611 | UK | 2019/7/1 |  |  | 0 |  |
| GCA_019480705.1 | strain: 818892 | UK | 2019/10/3 |  |  | 0 |  |
| GCA_019516785.1 | strain: 666455 | UK | 2019/1/1 |  |  | 0 |  |
| GCA_019531235.1 | strain: 1182232 | UK | 2021/5/1 |  |  | 0 |  |
| GCA_019531435.1 | strain: 1182249 | UK | 2021/5/1 |  |  | 0 |  |
| GCA_019532855.1 | strain: 1182187 | UK | 2021/5/2 |  |  | 0 |  |
| GCA_019536835.1 | strain: 1182227 | UK | 2021/5/3 |  | 2 | 0 | *bla*_TEM-1B_1_*, tet*(B)*_2* |
| GCA_019537475.1 | strain: 1182266 | UK | 2021/5/4 |  |  | 0 |  |
| GCA_019537835.1 | strain: 1182254 | UK | 2021/5/5 |  |  | 0 |  |
| GCA_019537855.1 | strain: 1182259 | UK | 2021/5/6 |  |  | 0 |  |
| GCA_019537955.1 | strain: 1182241 | UK | 2021/5/7 | 1 | 4 | 1 | *bla*_TEM-1B_1_*, bla*_DHA-1_1_*, tet*(A)*, mph*(A)*_2, qnrB4_1* |
| GCA_019538795.1 | strain: 1182195 | UK | 2021/5/8 |  |  | 0 |  |
| GCA_019538955.1 | strain: 1182234 | UK | 2021/5/9 |  |  | 0 |  |
| GCA_019548675.1 | strain: 561087 | UK | 2018/6/1 |  |  | 0 |  |
| GCA_019554595.1 | strain: 604915 | UK | 2018/9/1 |  |  | 0 |  |
| GCA_019574735.1 | strain: 1306298 | UK | 2021/7/1 |  |  | 0 |  |
| GCA_019702345.1 | strain: 1324247 | UK | 2021/8/1 |  |  | 0 |  |
| GCA_019941615.1 | strain: 1337763 | UK | 2021/8/2 |  |  | 0 |  |
| GCA_020463185.1 | strain: 1489925 | UK | 2021/9/1 |  |  | 0 |  |
| GCA_022764805.1 | strain: CNTRL_EPEC46 | demmark | - |  |  | 0 |  |
| GCA_023102595.1 | strain: 1617683 | UK | 2022/4/1 | 1 | 3 | 1 | *bla*_TEM-1B_1_*, bla*_DHA-1_1_*, tet*(A)*, qnrB4_1* |
| GCA_023830615.1 | strain: 1643233 | UK | 2022/5/1 |  |  | 0 |  |
| GCA_024007615.1 | strain: 1650191 | UK | 2022/6/1 |  |  | 0 |  |
| GCA_024112315.1 | strain: 1638548 | UK | 2022/5/1 | 1 | 4 | 1 | *bla*_TEM-1B_1_*, bla*_DHA-1_1_*, tet*(A)*, mph*(A)*_2, qnrB4_1* |
| GCA_024289115.1 | strain: 1661437 | UK | 2022/7/1 |  |  | 0 |  |
| GCA_024300365.1 | strain: 1664262 | UK | 2022/7/1 |  |  | 0 |  |
| GCA_024304085.1 | strain: 1662757 | UK | 2022/7/1 |  |  | 0 |  |
| GCA_024394735.1 | strain: 1669996 | UK | 2022/7/1 |  |  | 0 |  |
| GCA_024543305.1 | strain: 1672429 | UK | 2022/9/1 |  |  | 0 |  |
| GCA_024846805.1 | strain: 1688432 | UK | 2022/8/1 |  | 1 | 0 | *bla*_TEM-216_ |
| GCA_024950485.1 | strain: 1682252 | UK | 2022/8/1 |  |  | 0 |  |
| GCA_902387835.1 | isolate: MGYG-HGUT-02507 | USA | - |  |  | 0 |  |
| SRR13494855 | ESA011;Sample 41; | China | 2015 |  |  | 0 |  |
| SRR13494854 | ESA012;Sample 42; | China | 2015 |  | 4 | 1 | *tet*(A)*, oqxA_1, oqxB_1, aph(6)-Id_1, aph(3')-Ia_3, aph(3'')-Ib_5, aac(3)-IId_1, sul2* |
| SRR13494857 | ESA010;Sample 40; | China | 2015 |  |  | 0 |  |
| SRR13494865 | ESA002;Sample 32; | China | 2014 |  | 1 | 0 | *tet*(A) |
| SRR13494864 | ESA003;Sample 33; | China | 2014 |  |  | 0 |  |
| SAMN48536449 | ESA045 | China |  | 1 | 6 | 1 | *floR_2, bla*_CTX-M-55_1_*, tet*(A)*, oqxA_1, oqxB_1, aph(6)-Id_1, aph(3'')-Ib_5, aph(3')-IIa_2, sul2* |
| SAMN48536450 | ESA046 | China | 2014 |  |  | 0 |  |
| SAMN48536451 | ESA304 | China | 2023 | 1 | 6 | 1 | *floR_2, bla*_TEM-176_1_*, tet*(A)*, qnrS1, aph(3')-Ia_3, dfrA14_5* |
| [SRR25224797](https://www.ncbi.nlm.nih.gov/sra/SRR25224797) | ESA305 | China | 2023/02 |  |  | 0 |  |
| [SRR25224796](https://www.ncbi.nlm.nih.gov/sra/SRR25224797) | ESA306 | China | 2023/02 |  |  | 0 |  |
| [SRR25224795](https://www.ncbi.nlm.nih.gov/sra/SRR25224797) | ESA307 | China | 2023/02 |  |  | 0 |  |
| SRR252247924 | ESA308 | China | 2023/02 |  |  | 0 |  |
| SRR252247923 | ESA309 | China | 2023/02 |  |  | 0 |  |
| SRR25224792 | strain: DCY512; ESA310 | China | 2023 |  |  | 0 |  |
| CP157783-CP157791 | ESA311 | China | 2023/08/17 | 1 | 8 | 1 | *cmlA1, floR_2, bla*_OXA-1_1_*, bla*_TEM-1B_1_*, tet*(A)*, mef*(B)*, mph*(A)*_2, qnrS1, aph(4)-Ia, aac(3)-IId_1, aac(3)-Ⅳa_1, aac(6')-Ib-cr_1, aadA2_1, aadA5_1, ant(3'')-Ia_1, dfrA12, dfrA17, sul2, sul3* |
| [GCA_040114695.1](https://www.ncbi.nlm.nih.gov/datasets/genome/GCA_040114695.1/) | ESA339 | China | 2023/9/15 |  | 4 | 1 | *cmlA1, floR_2, tet*(A)*, qnrS1, aph(3')-Ia_3* |
| SAMN48536452 | ESA350 | China | 2023/12/3 | 1 | 9 | 1 | *floR_2, bla*_TEM-1B_1_*, bla*_CTX-M-55_1_*, tet*(A)*, mph*(A)*_2, qnrS1, aph(6)-Id_1, aph(3')-Ia_3, aac(3)-IIa_1, aac(3)-IId_1, aadA2_1, ant(3'')-Ia_1, ARR-3_4, dfrA12, sul3, lnu*(F)*_1* |
| SAMN48536453 | ESA353 | China | 2024/8/27 |  | 3 | 1 | *bla*_CTX-M-14_1_*, tet*(A)*, aph(6)-Id_1, aac(3)-IIa_1* |
| CP136258-CP136262 | strain: ESA302 | China | 2014 | 1 | 6 | 1 | *floR_2, bla*_CTX-M-55_1_*, tet*(A)*, oqxB_1, aph(6)-Id_1, aph(3'')-Ib_5, aph(3')-IIa_2, sul2* |
| GCA_030410295.1 | strain: ESA303 | China | 2022 |  |  | 0 |  |
| GCA_016904755.2 | strain: Sample 167; ESA138 | China | 2018 |  |  | 0 |  |

¹ Number of predicted drug classes;

² MDR status was defined as resistance to >=3 antibiotic classes.1="yes",0="No".


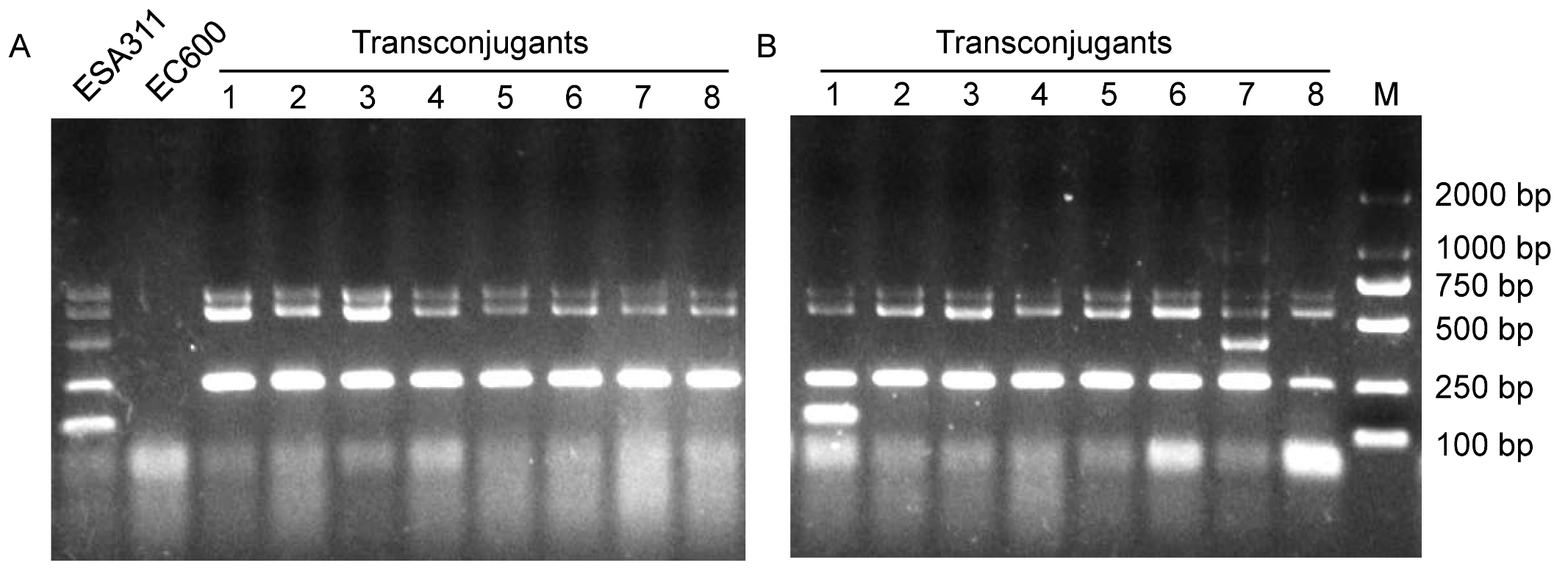


**Figure S1. Confirmation of plasmid presence in transconjugants by PCR analysis.** (A) PCR amplification of *repA* genes from donor strain *E.albertii* ESA311, recipient strain *E. coli* EC600, and transconjugants selected on LB agar plates supplemented with 50 μg/mL chloramphenicol and 100 μg/mL rifampin. Lane ESA311: repA-specific PCR products from donor strain ESA311 showing five distinct bands corresponding to plasmids (by descending size): pESA311_5 (650 bp), pESA311_1 (557 bp), pESA311_4 (416 bp), pESA311_2 (261 bp), and pESA311_3 (134 bp). Lane EC600: No amplification products observed in the plasmid-free recipient strain EC600, confirming primer specificity. Lanes 1-8: PCR results from transconjugants demonstrating successful plasmid transfer. (B) PCR analysis of transconjugants selected on LB agar plates supplemented with 2 μg/mL ciprofloxacin and 100 μg/mL ceftazidime. Lanes 1–8: PCR results from transconjugants under alternative antibiotic selection. Lane M: DNA ladder (100–2000 bp; GeneRuler™, Thermo Scientific). All primers used are listed in Table S1.
